# Supplementary material for: Does probe-tube verification of real-ear hearing aid amplification characteristics improve outcomes in adult hearing aid users? A protocol for a systematic review
Source: BMJ Open. 2020 Jul 19;10(7):e038113. doi: 10.1136/bmjopen-2020-038113 (PMC7371126; doi:10.1136/bmjopen-2020-038113)
Supplement: Supplementary data [file bmjopen-2020-038113supp001.pdf]

Appendix 1: Search Strategies

A. COCHRANE Library

(("Hearing Loss" OR "hearing loss" OR "hearing losses" OR deaf* OR "Hearing problem" OR "hearing problems" OR hypoacus* OR "hearing impaired" OR "hearing impairment" OR "hearing impairments" OR "hard of hearing" OR "hearing difficulties" OR "hearing difficulty" OR "hearing disabilities" OR "hearing disability" OR "Hearing Disorders" OR "hearing disorder" OR "hearing disorders" OR "hearing loss*" OR "Hearing problem*" OR "hearing impair*" OR "hearing difficult*" OR "hearing disab*" OR "hearing disorder*") AND ("Hearing Aids" OR "hearing aid" OR "hearing aids" OR "hearing device" OR "hearing devices" OR "acoustic aid" OR "acoustic aids" OR "ear molds" OR "ear mold" OR "HA" OR "aid") AND ("real ear measurement" OR "real ear measurements" OR "real ear measure" OR "real ear measures" OR "real-ear measurement" OR "real-ear measurements" OR "real-ear measure" OR "real-ear measures" OR "REM" OR PMM* OR "probe microphone" OR "probe microphones" OR "probe mic" OR "probe mics" OR "probe microphone measurement" OR "probe microphone measurements" OR "probe microphone measure" OR "probe microphone measures" OR "probe-microphone" OR "probe-microphones" OR "probe-microphone measurement" OR "probe-microphone measurements" OR "probe-microphone measure" OR "probe-microphone measures" OR "initial fit" OR "initial fits" OR "initial fitting" OR "initial fittings" OR "real ear" OR "real ears" OR "real-ear" OR "real-ears" OR "hearing aid fit" OR "hearing aid fitter" OR "hearing aid fitting" OR "hearing aid fittings")):ti,ab,kw NOT (("Child" OR "Infant") NOT "Adult*"):ti

B. Embase

(((exp *"Hearing Aid"/ OR "hearing aid".ti OR "hearing aids".ti OR "hearing device".ti OR "hearing devices".ti OR "acoustic aid".ti OR "acoustic aids".ti OR "ear molds".ti OR "ear mold".ti OR "HA".ti OR "aid".ti) AND ("real ear measurement".ti OR "real ear measurements".ti OR "real ear measure".ti OR "real ear measures".ti OR "real-ear measurement".ti OR "real-ear measurements".ti OR "real-ear measure".ti OR "real-ear measures".ti OR "REM".ti OR PMM*.ti OR "probe microphone".ti OR "probe microphones".ti OR "probe mic".ti OR "probe mics".ti OR "probe microphone measurement".ti OR "probe microphone measurements".ti OR "probe microphone measure".ti OR "probe microphone measures".ti OR "probe-microphone".ti OR "probe-microphones".ti OR "probe-microphone measurement".ti OR "probe-microphone measurements".ti OR "probe-microphone measure".ti OR "probe-microphone measures".ti OR "initial fit".ti OR "initial fits".ti OR "initial fitting".ti OR "initial fittings".ti OR "real ear".ti OR "real ears".ti OR "real-ear".ti OR "real-ears".ti OR "hearing aid fit".ti OR "hearing aid fitter".ti OR "hearing aid fitting".ti OR "hearing aid fittings".ti) NOT (exp "juvenile"/ NOT exp "adult"/) NOT (conference review or conference abstract).pt NOT ("case report"/ OR ((exp "review"/ OR exp "Practice Guideline"/ OR "guideline".ti OR "guidelines".ti) NOT exp "Clinical Study"/))) OR ((exp *"Hearing Aid"/ OR "hearing aid".ti,ab OR "hearing

aids".ti,ab OR "hearing device".ti,ab OR "hearing devices".ti,ab OR "acoustic aid".ti,ab OR "acoustic aids".ti,ab OR "ear molds".ti,ab OR "ear mold".ti,ab OR "HA".ti,ab OR "aid".ti,ab) AND ("real ear measurement".mp OR "real ear measurements".mp OR "real ear measure".mp OR "real ear measures".mp OR "real-ear measurement".mp OR "real-ear measurements".mp OR "real-ear measure".mp OR "real-ear measures".mp OR "REM".mp OR PMM*.mp OR "probe microphone".mp OR "probe microphones".mp OR "probe mic".mp OR "probe mics".mp OR "probe microphone measurement".mp OR "probe microphone measurements".mp OR "probe microphone measure".mp OR "probe microphone measures".mp OR "probe-microphone".mp OR "probe-microphones".mp OR "probe-microphone measurement".mp OR "probe-microphone measurements".mp OR "probe-microphone measure".mp OR "probe-microphone measures".mp OR "initial fit".mp OR "initial fits".mp OR "initial fitting".mp OR "initial fittings".mp OR "real ear".mp OR "real ears".mp OR "real-ear".mp OR "real-ears".mp OR "hearing aid fit".mp OR "hearing aid fitter".mp OR "hearing aid fitting".mp OR "hearing aid fittings".mp) AND (exp *"Hearing Impairment"/ OR "hearing loss".ti,ab OR "hearing losses".ti,ab OR deaf*.ti,ab OR "Hearing problem".ti,ab OR "hearing problems".ti,ab OR hypoacus*.ti,ab OR "hearing impaired".ti,ab OR "hearing impairment".ti,ab OR "hearing impairments".ti,ab OR "hard of hearing".ti,ab OR "hearing difficulties".ti,ab OR "hearing difficulty".ti,ab OR "hearing disabilities".ti,ab OR "hearing disability".ti,ab OR "hearing loss*".ti,ab OR "Hearing problem*".ti,ab OR "hearing impair*".ti,ab OR "hearing difficult*".ti,ab OR "hearing disab*".ti,ab OR "hearing disorder*".ti,ab) AND ("randomised".mp OR "randomized".mp OR "nonrandomised".mp OR "nonrandomised".mp OR exp "Randomization"/ OR "Randomized Controlled Trial"/ OR exp "Controlled Clinical Trial"/ OR "Equivalence Trial"/ OR "Pragmatic Trial"/ OR "quasi experimental study"/ OR "Random Allocation".mp OR "Controlled Clinical Trial".mp OR "Controlled Clinical Trials".mp OR "Controlled Trial".mp OR "Controlled Trials".mp OR "Equivalence Trial".mp OR "Pragmatic Clinical Trial".mp OR "Equivalence Trials".mp OR "Pragmatic Clinical Trials".mp OR "RCT".mp OR "RCTs".mp OR "Quasi-Experimental".mp OR "Quasi-Experiment*".mp OR "QuasiExperimental".mp OR QuasiExperiment*.mp OR "Crossover Procedure"/ OR "Cross-Over".mp OR "CrossOver".mp OR "retrospective".mp OR Retrospectiv*.mp OR "Retrospective Study"/ OR "Prospective Study"/ OR "prospective".mp OR prospectiv*.mp OR exp "Evaluation Study"/ OR "evaluate".mp OR evaluat*.mp OR exp "Comparative Study"/ OR "compare".mp OR "comparative".mp OR compar*.mp) NOT (exp "juvenile"/ NOT exp "adult"/) NOT (conference review or conference abstract).pt NOT ("case report"/ OR ((exp "review"/ OR exp "Practice Guideline"/ OR "guideline".ti OR "guidelines".ti) NOT exp "Clinical Study"/))))

C. Emcare

(((exp *"Hearing Aid"/ OR "hearing aid".ti OR "hearing aids".ti OR "hearing device".ti OR "hearing devices".ti OR "acoustic aid".ti OR "acoustic aids".ti OR "ear molds".ti OR "ear mold".ti OR "HA".ti OR "aid".ti) AND ("real ear measurement".ti OR "real ear measurements".ti OR "real ear measure".ti OR "real ear measures".ti OR "real-ear measurement".ti OR "real-ear measurements".ti OR "real-ear measure".ti OR "real-ear

measures".ti OR "REM".ti OR PMM*.ti OR "probe microphone".ti OR "probe microphones".ti OR "probe mic".ti OR "probe mics".ti OR "probe microphone measurement".ti OR "probe microphone measurements".ti OR "probe microphone measure".ti OR "probe microphone measures".ti OR "probe-microphone".ti OR "probe-microphones".ti OR "probe-microphone measurement".ti OR "probe-microphone measurements".ti OR "probe-microphone measure".ti OR "probe-microphone measures".ti OR "initial fit".ti OR "initial fits".ti OR "initial fitting".ti OR "initial fittings".ti OR "real ear".ti OR "real ears".ti OR "real-ear".ti OR "real-ears".ti OR "hearing aid fit".ti OR "hearing aid fitter".ti OR "hearing aid fitting".ti OR "hearing aid fittings".ti) NOT (exp "juvenile"/ NOT exp "adult"/) NOT (conference review or conference abstract).pt NOT ("case report"/ OR ((exp "review"/ OR exp "Practice Guideline"/ OR "guideline".ti OR "guidelines".ti) NOT exp "Clinical Study"/))) OR ((exp *"Hearing Aid"/ OR "hearing aid".ti,ab OR "hearing aids".ti,ab OR "hearing device".ti,ab OR "hearing devices".ti,ab OR "acoustic aid".ti,ab OR "acoustic aids".ti,ab OR "ear molds".ti,ab OR "ear mold".ti,ab OR "HA".ti,ab OR "aid".ti,ab) AND ("real ear measurement".mp OR "real ear measurements".mp OR "real ear measure".mp OR "real ear measures".mp OR "real-ear measurement".mp OR "real-ear measurements".mp OR "real-ear measure".mp OR "real-ear measures".mp OR "REM".mp OR PMM*.mp OR "probe microphone".mp OR "probe microphones".mp OR "probe mic".mp OR "probe mics".mp OR "probe microphone measurement".mp OR "probe microphone measurements".mp OR "probe microphone measure".mp OR "probe microphone measures".mp OR "probe-microphone".mp OR "probe-microphones".mp OR "probe-microphone measurement".mp OR "probe-microphone measurements".mp OR "probe-microphone measure".mp OR "probe-microphone measures".mp OR "initial fit".mp OR "initial fits".mp OR "initial fitting".mp OR "initial fittings".mp OR "real ear".mp OR "real ears".mp OR "real-ear".mp OR "real-ears".mp OR "hearing aid fit".mp OR "hearing aid fitter".mp OR "hearing aid fitting".mp OR "hearing aid fittings".mp) AND (exp *"Hearing Impairment"/ OR "hearing loss".ti,ab OR "hearing losses".ti,ab OR deaf*.ti,ab OR "Hearing problem".ti,ab OR "hearing problems".ti,ab OR hypoacus*.ti,ab OR "hearing impaired".ti,ab OR "hearing impairment".ti,ab OR "hearing impairments".ti,ab OR "hard of hearing".ti,ab OR "hearing difficulties".ti,ab OR "hearing difficulty".ti,ab OR "hearing disabilities".ti,ab OR "hearing disability".ti,ab OR "hearing loss*".ti,ab OR "Hearing problem*".ti,ab OR "hearing impair*".ti,ab OR "hearing difficult*".ti,ab OR "hearing disab*".ti,ab OR "hearing disorder*".ti,ab) AND ("randomised".mp OR "randomized".mp OR "nonrandomised".mp OR "nonrandomised".mp OR exp "Randomization"/ OR "Randomized Controlled Trial"/ OR exp "Controlled Clinical Trial"/ OR "Equivalence Trial"/ OR "Pragmatic Trial"/ OR "quasi experimental study"/ OR "Random Allocation".mp OR "Controlled Clinical Trial".mp OR "Controlled Clinical Trials".mp OR "Controlled Trial".mp OR "Controlled Trials".mp OR "Equivalence Trial".mp OR "Pragmatic Clinical Trial".mp OR "Equivalence Trials".mp OR "Pragmatic Clinical Trials".mp OR "RCT".mp OR "RCTs".mp OR "Quasi-Experimental".mp OR "Quasi-Experiment*".mp OR "QuasiExperimental".mp OR QuasiExperiment*.mp OR "Crossover Procedure"/ OR "Cross-Over".mp OR "CrossOver".mp OR "retrospective".mp OR Retrospectiv*.mp OR "Retrospective Study"/ OR "Prospective Study"/ OR "prospective".mp OR prospectiv*.mp OR exp "Evaluation Study"/ OR "evaluate".mp OR evaluat*.mp OR exp "Comparative Study"/ OR "compare".mp OR "comparative".mp OR

compar*.mp) NOT (exp "juvenile"/ NOT exp "adult"/) NOT (conference review or conference abstract).pt NOT ("case report"/ OR ((exp "review"/ OR exp "Practice Guideline"/ OR "guideline".ti OR "guidelines".ti) NOT exp "Clinical Study"/))))

D. MEDLINE

(("Hearing Loss"/ OR "hearing loss".mp OR "hearing losses".mp OR deaf*.mp OR "Hearing problem".mp OR "hearing problems".mp OR hypoacus*.mp OR "hearing impaired".mp OR "hearing impairment".mp OR "hearing impairments".mp OR "hard of hearing".mp OR "hearing difficulties".mp OR "hearing difficulty".mp OR "hearing disabilities".mp OR "hearing disability".mp OR exp "Hearing Disorders"/ OR "hearing disorder".mp OR "hearing disorders".mp OR hearing loss*.mp OR Hearing problem*.mp OR hearing impair*.mp OR hearing difficult*.mp OR hearing disab*.mp OR hearing disorder*.mp) AND (exp "Hearing Aids"/ OR "hearing aid".mp OR "hearing aids".mp OR "hearing device".mp OR "hearing devices".mp OR "acoustic aid".mp OR "acoustic aids".mp OR "ear molds".mp OR "ear mold".mp OR "HA".ti,ab OR "aid".mp) AND ("real ear measurement".mp OR "real ear measurements".mp OR "real ear measure".mp OR "real ear measures".mp OR "real-ear measurement".mp OR "real-ear measurements".mp OR "real-ear measure".mp OR "real-ear measures".mp OR "REM".mp OR PMM*.mp OR "probe microphone".mp OR "probe microphones".mp OR "probe mic".mp OR "probe mics".mp OR "probe microphone measurement".mp OR "probe microphone measurements".mp OR "probe microphone measure".mp OR "probe microphone measures".mp OR "probe-microphone".mp OR "probe-microphones".mp OR "probe-microphone measurement".mp OR "probe-microphone measurements".mp OR "probe-microphone measure".mp OR "probe-microphone measures".mp OR "initial fit".mp OR "initial fits".mp OR "initial fitting".mp OR "initial fittings".mp OR "real ear".mp OR "real ears".mp OR "real-ear".mp OR "real-ears".mp OR "hearing aid fit".mp OR "hearing aid fitter".mp OR "hearing aid fitting".mp OR "hearing aid fittings".mp) AND ("randomised".mp OR "randomized".mp OR "nonrandomised".mp OR "nonrandomised".mp OR "Random Allocation"/ OR exp "Randomized Controlled Trial"/ OR exp "Randomized Controlled Trials as Topic"/ OR "Non-Randomized Controlled Trials as Topic"/ OR exp "Controlled Clinical Trial"/ OR exp "Equivalence Trial"/ OR exp "Pragmatic Clinical Trial"/ OR exp "Equivalence Trials as Topic"/ OR exp "Pragmatic Clinical Trials as Topic"/ OR "Random Allocation".mp OR "Controlled Clinical Trial".mp OR "Controlled Clinical Trials".mp OR "Controlled Trial".mp OR "Controlled Trials".mp OR "Equivalence Trial".mp OR "Pragmatic Clinical Trial".mp OR "Equivalence Trials".mp OR "Pragmatic Clinical Trials".mp OR "RCT".mp OR "RCTs".mp OR "Quasi-Experimental".mp OR Quasi-Experiment*.mp OR "QuasiExperimental".mp OR QuasiExperiment*.mp OR "Cross-Over Studies"/ OR "Cross-Over".mp OR "CrossOver".mp OR "retrospective".mp OR Retrospectiv*.mp OR exp "Retrospective Studies"/ OR "Prospective Studies"/ OR "prospective".mp OR prospectiv*.mp OR exp "Evaluation Study"/ OR exp "Evaluation Studies as Topic"/ OR "evaluate".mp OR evaluat*.mp OR exp "Comparative Study"/ OR "compare".mp OR "comparative".mp OR compar*.mp) NOT ((exp "Child"/ OR exp "Infant"/) NOT exp "Adult"/) NOT (("Case Reports"/ OR "case report".ti OR "Review"/ OR

"review".ti OR exp "Guideline"/ OR "guideline".ti OR "guidelines".ti) NOT (exp "Clinical Study"/ OR "trial".ti OR "RCT".ti)))

E. PsycINFO

(TX("Hearing Loss" OR "hearing loss" OR "hearing losses" OR deaf* OR "Hearing problem" OR "hearing problems" OR hypoacus* OR "hearing impaired" OR "hearing impairment" OR "hearing impairments" OR "hard of hearing" OR "hearing difficulties" OR "hearing difficulty" OR "hearing disabilities" OR "hearing disability" OR "Hearing Disorders" OR "hearing disorder" OR "hearing disorders" OR "hearing loss*" OR "Hearing problem*" OR "hearing impair*" OR "hearing difficult*" OR "hearing disab*" OR "hearing disorder*") AND TX("Hearing Aids" OR "hearing aid" OR "hearing aids" OR "hearing device" OR "hearing devices" OR "acoustic aid" OR "acoustic aids" OR "ear molds" OR "ear mold" OR "HA" OR "aid") AND TX("real ear measurement" OR "real ear measurements" OR "real ear measure" OR "real ear measures" OR "real-ear measurement" OR "real-ear measurements" OR "real-ear measure" OR "real-ear measures" OR "REM" OR PMM* OR "probe microphone" OR "probe microphones" OR "probe mic" OR "probe mics" OR "probe microphone measurement" OR "probe microphone measurements" OR "probe microphone measure" OR "probe microphone measures" OR "probe-microphone" OR "probe-microphones" OR "probe-microphone measurement" OR "probe-microphone measurements" OR "probe-microphone measure" OR "probe-microphone measures" OR "initial fit" OR "initial fits" OR "initial fitting" OR "initial fittings" OR "real ear" OR "real ears" OR "real-ear" OR "real-ears" OR "hearing aid fit" OR "hearing aid fitter" OR "hearing aid fitting" OR "hearing aid fittings") AND TX("randomised" OR "randomized" OR "nonrandomised" OR "nonrandomised" OR "Random Allocation" OR "Randomized Controlled Trial" OR "Randomized Controlled Trials as Topic" OR "Non-Randomized Controlled Trials as Topic" OR "Controlled Clinical Trial" OR "Equivalence Trial" OR "Pragmatic Clinical Trial" OR "Equivalence Trials as Topic" OR "Pragmatic Clinical Trials as Topic" OR "Random Allocation" OR "Controlled Clinical Trial" OR "Controlled Clinical Trials" OR "Controlled Trial" OR "Controlled Trials" OR "Equivalence Trial" OR "Pragmatic Clinical Trial" OR "Equivalence Trials" OR "Pragmatic Clinical Trials" OR "RCT" OR "RCTs" OR "Quasi-Experimental" OR Quasi-Experiment* OR "QuasiExperimental" OR QuasiExperiment* OR "Cross-Over Studies" OR "Cross-Over" OR "CrossOver" OR "retrospective" OR Retrospectiv* OR "Retrospective Studies" OR "Prospective Studies" OR "prospective" OR prospectiv* OR "Evaluation Study" OR "Evaluation Studies as Topic" OR "evaluate" OR evaluat* OR "Comparative Study" OR "compare" OR "comparative" OR compar*) NOT TI(("Child" OR "Infant") NOT "Adult") NOT TI(("Case Reports" OR "case report" OR "Review" OR "review" OR "Guideline" OR "guideline" OR "guidelines") NOT ("Clinical Study" OR "trial" OR "RCT")))

F. PubMed

(("Hearing Loss"[Mesh] OR "hearing loss"[tw] OR "hearing losses"[tw] OR deaf*[tw] OR "Hearing problem"[tw] OR "hearing problems"[tw] OR hypoacus*[tw] OR "hearing impaired"[tw] OR "hearing impairment"[tw] OR "hearing impairments"[tw] OR "hard of hearing"[tw] OR "hearing difficulties"[tw] OR "hearing difficulty"[tw] OR "hearing disabilities"[tw] OR "hearing disability"[tw] OR "Hearing Disorders"[mesh] OR "hearing disorder"[tw] OR "hearing disorders"[tw] OR hearing loss*[tw] OR Hearing problem*[tw] OR hearing impair*[tw] OR hearing difficult*[tw] OR hearing disab*[tw] OR hearing disorder*[tw]) AND ("Hearing Aids"[Mesh] OR "hearing aid"[tw] OR "hearing aids"[tw] OR "hearing device"[tw] OR "hearing devices"[tw] OR "acoustic aid"[tw] OR "acoustic aids"[tw] OR "ear molds"[tw] OR "ear mold"[tw] OR "HA"[tiab] OR "aid"[tw]) AND ("real ear measurement"[tw] OR "real ear measurements"[tw] OR "real ear measure"[tw] OR "real ear measures"[tw] OR "real-ear measurement"[tw] OR "real-ear measurements"[tw] OR "real-ear measure"[tw] OR "real-ear measures"[tw] OR "REM"[tw] OR PMM*[tw] OR "probe microphone"[tw] OR "probe microphones"[tw] OR "probe mic"[tw] OR "probe mics"[tw] OR "probe microphone measurement"[tw] OR "probe microphone measurements"[tw] OR "probe microphone measure"[tw] OR "probe microphone measures"[tw] OR "probe-microphone"[tw] OR "probe-microphones"[tw] OR "probe-microphone measurement"[tw] OR "probe-microphone measurements"[tw] OR "probe-microphone measure"[tw] OR "probe-microphone measures"[tw] OR "initial fit"[tw] OR "initial fits"[tw] OR "initial fitting"[tw] OR "initial fittings"[tw] OR "real ear"[tw] OR "real ears"[tw] OR "real-ear"[tw] OR "real-ears"[tw] OR "hearing aid fit"[tw] OR "hearing aid fitter"[tw] OR "hearing aid fitting"[tw] OR "hearing aid fittings"[tw]) AND ("randomised"[tw] OR "randomized"[tw] OR "nonrandomised"[tw] OR "nonrandomised"[tw] OR "Random Allocation"[Mesh] OR "Randomized Controlled Trial"[Ptyp] OR "Randomized Controlled Trials as Topic"[Mesh] OR "Non-Randomized Controlled Trials as Topic"[Mesh] OR "Controlled Clinical Trial"[ptyp] OR "Equivalence Trial"[ptyp] OR "Pragmatic Clinical Trial"[ptyp] OR "Equivalence Trials as Topic"[Mesh] OR "Pragmatic Clinical Trials as Topic"[Mesh] OR "Random Allocation"[tw] OR "Controlled Clinical Trial"[tw] OR "Controlled Clinical Trials"[tw] OR "Controlled Trial"[tw] OR "Controlled Trials"[tw] OR "Equivalence Trial"[tw] OR "Pragmatic Clinical Trial"[tw] OR "Equivalence Trials"[tw] OR "Pragmatic Clinical Trials"[tw] OR "RCT"[tw] OR "RCTs"[tw] OR "Quasi-Experimental"[tw] OR Quasi-Experiment*[tw] OR "QuasiExperimental"[tw] OR QuasiExperiment*[tw] OR "Cross-Over Studies"[Mesh] OR "Cross-Over"[tw] OR "CrossOver"[tw] OR "retrospective"[tw] OR Retrospectiv*[tw] OR "Retrospective Studies"[Mesh] OR "Prospective Studies"[Mesh] OR "prospective"[tw] OR prospectiv*[tw] OR "Evaluation Study"[ptyp] OR "Evaluation Studies as Topic"[Mesh] OR "evaluate"[tw] OR evaluat*[tw] OR "Comparative Study"[ptyp] OR "compare"[tw] OR "comparative"[tw] OR compar*[tw]) NOT (("Child"[mesh] OR "Infant"[mesh]) NOT "Adult"[mesh]) NOT (("Case Reports"[ptyp] OR "case report"[ti] OR "Review"[ptyp] OR "review"[ti] OR "Guideline"[Publication Type] OR "guideline"[ti] OR "guidelines"[ti]) NOT ("Clinical Study"[ptyp] OR "trial"[ti] OR "RCT"[ti])))

G. Web of Science

(ts=("Hearing Loss" OR "hearing loss" OR "hearing losses" OR deaf* OR "Hearing problem" OR "hearing problems" OR hypoacus* OR "hearing impaired" OR "hearing impairment" OR "hearing impairments" OR "hard of hearing" OR "hearing difficulties" OR "hearing difficulty" OR "hearing disabilities" OR "hearing disability" OR "Hearing Disorders" OR "hearing disorder" OR "hearing disorders" OR "hearing loss*" OR "Hearing problem*" OR "hearing impair*" OR "hearing difficult*" OR "hearing disab*" OR "hearing disorder*") AND ts=("Hearing Aids" OR "hearing aid" OR "hearing aids" OR "hearing device" OR "hearing devices" OR "acoustic aid" OR "acoustic aids" OR "ear molds" OR "ear mold" OR "HA" OR "aid") AND ts=("real ear measurement" OR "real ear measurements" OR "real ear measure" OR "real ear measures" OR "real-ear measurement" OR "real-ear measurements" OR "real-ear measure" OR "real-ear measures" OR "REM" OR PMM* OR "probe microphone" OR "probe microphones" OR "probe mic" OR "probe mics" OR "probe microphone measurement" OR "probe microphone measurements" OR "probe microphone measure" OR "probe microphone measures" OR "probe-microphone" OR "probe-microphones" OR "probe-microphone measurement" OR "probe-microphone measurements" OR "probe-microphone measure" OR "probe-microphone measures" OR "initial fit" OR "initial fits" OR "initial fitting" OR "initial fittings" OR "real ear" OR "real ears" OR "real-ear" OR "real-ears" OR "hearing aid fit" OR "hearing aid fitter" OR "hearing aid fitting" OR "hearing aid fittings") AND ts=("randomised" OR "randomized" OR "nonrandomised" OR "nonrandomised" OR "Random Allocation" OR "Randomized Controlled Trial" OR "Randomized Controlled Trials as Topic" OR "Non-Randomized Controlled Trials as Topic" OR "Controlled Clinical Trial" OR "Equivalence Trial" OR "Pragmatic Clinical Trial" OR "Equivalence Trials as Topic" OR "Pragmatic Clinical Trials as Topic" OR "Random Allocation" OR "Controlled Clinical Trial" OR "Controlled Clinical Trials" OR "Controlled Trial" OR "Controlled Trials" OR "Equivalence Trial" OR "Pragmatic Clinical Trial" OR "Equivalence Trials" OR "Pragmatic Clinical Trials" OR "RCT" OR "RCTs" OR "Quasi-Experimental" OR Quasi-Experiment* OR "QuasiExperimental" OR QuasiExperiment* OR "Cross-Over Studies" OR "Cross-Over" OR "CrossOver" OR "retrospective" OR Retrospectiv* OR "Retrospective Studies" OR "Prospective Studies" OR "prospective" OR prospectiv* OR "Evaluation Study" OR "Evaluation Studies as Topic" OR "evaluate" OR evaluat* OR "Comparative Study" OR "compare" OR "comparative" OR compar*) NOT ti=(("Child" OR "Infant") NOT "Adult") NOT ti=(("Case Reports" OR "case report" OR "Review" OR "review" OR "Guideline" OR "guideline" OR "guidelines") NOT ("Clinical Study" OR "trial" OR "RCT")) NOT dt=(meeting abstract))
