# Supplementary material for: Does probe-tube verification of real-ear hearing aid amplification characteristics improve outcomes in adult hearing aid users? A protocol for a systematic review
Source: BMJ Open. 2020 Jul 19;10(7):e038113. doi: 10.1136/bmjopen-2020-038113 (PMC7371126; doi:10.1136/bmjopen-2020-038113)
Supplement: Author's manuscript [file bmjopen-2020-038113.draft_revisions.pdf]

BMJ Open is committed to open peer review. As part of this commitment we make the peer review history of every article we publish publicly available.

When an article is published we post the peer reviewers' comments and the authors' responses online. We also post the versions of the paper that were used during peer review. These are the versions that the peer review comments apply to.

The versions of the paper that follow are the versions that were submitted during the peer review process. They are not the versions of record or the final published versions. They should not be cited or distributed as the published version of this manuscript.

BMJ Open is an open access journal and the full, final, typeset and author-corrected version of record of the manuscript is available on our site with no access controls, subscription charges or pay-per-view fees (http://bmjopen.bmj.com).

If you have any questions on BMJ Open's open peer review process please email info.bmjopen@bmj.com

BMJ Open

Does probe-tube verification of real-ear hearing aid amplification characteristics improve outcomes in adult hearing aid users: a protocol for a systematic review

Journal:	*BMJ Open*
Manuscript ID	bmjopen-2020-038113
Article Type:	Protocol
Date Submitted by the Author:	27-Feb-2020
Complete List of Authors:	Almufarrij, Ibrahim; The University of Manchester, Manchester Centre for Audiology and Deafness, School of Health Sciences, University of Manchester and the Manchester Academic Health Science Centre; King Saud University, Department of Rehabilitation Sciences<br>Munro, Kevin; The University of Manchester, Manchester Centre for Audiology and Deafness, School of Health Sciences, University of Manchester and the Manchester Academic Health Science Centre; Manchester University NHS Foundation Trust, Manchester Academic Health Science Centre<br>Dillon, Harvey; The University of Manchester, Manchester Centre for Audiology and Deafness, School of Health Sciences, University of Manchester and the Manchester Academic Health Science Centre; Macquarie University
Keywords:	Audiology < OTOLARYNGOLOGY, OTOLARYNGOLOGY, Neurotology < OTOLARYNGOLOGY

SCHOLARONE™
Manuscripts

I, the Submitting Author has the right to grant and does grant on behalf of all authors of the Work (as defined in the below author licence), an exclusive licence and/or a non-exclusive licence for contributions from authors who are: i) UK Crown employees; ii) where BMJ has agreed a CC-BY licence shall apply, and/or iii) in accordance with the terms applicable for US Federal Government officers or employees acting as part of their official duties; on a worldwide, perpetual, irrevocable, royalty-free basis to BMJ Publishing Group Ltd ("BMJ") its licensees and where the relevant Journal is co-owned by BMJ to the co-owners of the Journal, to publish the Work in this journal and any other BMJ products and to exploit all rights, as set out in our licence.

The Submitting Author accepts and understands that any supply made under these terms is made by BMJ to the Submitting Author unless you are acting as an employee on behalf of your employer or a postgraduate student of an affiliated institution which is paying any applicable article publishing charge ("APC") for Open Access articles. Where the Submitting Author wishes to make the Work available on an Open Access basis (and intends to pay the relevant APC), the terms of reuse of such Open Access shall be governed by a Creative Commons licence – details of these licences and which Creative Commons licence will apply to this Work are set out in our licence referred to above.

Other than as permitted in any relevant BMJ Author's Self Archiving Policies, I confirm this Work has not been accepted for publication elsewhere, is not being considered for publication elsewhere and does not duplicate material already published. I confirm all authors consent to publication of this Work and authorise the granting of this licence.

For peer review only

Does probe-tube verification of real-ear hearing aid amplification characteristics improve outcomes in adult hearing aid users: a protocol for a systematic review

Authors: Ibrahim Almufarrij[1, 2], Kevin J Munro[1, 3] and Harvey Dillon[1,4]

Author affiliations

[1] Manchester Centre for Audiology and Deafness, School of Health Sciences, University of Manchester and the Manchester Academic Health Science Centre, UK

[2] Department of Rehabilitation Sciences, College of Applied Medical Sciences, King Saud University, Riyadh, Kingdom of Saudi Arabia

[3] Manchester University Hospitals NHS Foundation Trust, Manchester Academic Health Science Centre, UK

[4] Macquarie University, Sydney, Australia

Corresponding author: Ibrahim Almufarrij, Manchester Centre for Audiology and Deafness, University of Manchester, Manchester, M13 9PL, United Kingdom. E-mail: Email: ialmufarrij@ksu.edu.sa

For peer review only

ABSTRACT

Introduction: Using a probe-tube microphone to measure and adjust the real-ear performance of the hearing aid to match the prescription target is recommended and widely used in clinical practice. Hearing aid fitting software can approximately match the amplification characteristics of the hearing aid to the prescription without real-ear measurements (REM), but using REM improves the match to the prescribed target. What is unclear is if the improved match results in a better patient-reported outcome. The primary objective of this review is to determine whether the use of REM improves patient-reported outcomes in adult hearing aid users.

Methods and analysis: The review's methods are in accordance with Preferred Reporting Items for Systematic Reviews and Meta-Analyses (PRISMA-P) guidelines. MEDLINE, EMBASE, PsycINFO, CINAHL, Web of Science and CENTRAL via Cochrane Library will be searched to identify relevant studies. The review's population of interest will include adults with any degree of sensorineural or mixed hearing loss who have been prescribed with acoustic hearing aids. The included studies should compare REM fitting to the initial fit provided by the manufacturer's fitting software. Hearing-specific quality of life is the primary outcome but secondary outcomes include self-reported listening ability, speech recognition scores, generic quality of life, hours of use, number of required follow-up sessions and adverse events. Randomised and non-randomised controlled trials will be included. The risk of bias in the included studies will be evaluated using Down and Black's checklist. The quality of the overall evidence will be assessed using the Grading of Recommendations, Assessment, Development and Evaluations (GRADE) tool.

Ethics and dissemination: Ethical approval will not be sought because this systematic review will only retrieve and analyse data from published studies. Review results will be published in a peer-reviewed journal and presented at relevant scientific conferences.

PROSPERO registration number: CRD42020166074

For peer review only

Strengths and limitations of the study:

- This review will be first to synthesise data that compare two commonly used approaches to fit hearing aids to prescription targets (i.e. initial-fit and real-ear measurements).
- A variety of outcome measures will be used to compare these two approaches.
- The implications of this review are important for the emerging category of over-the-counter and direct-to-consumer hearing aids that do not involve real-ear measures.
- We will include only studies that compare these fitting approaches among adults with sensorineural hearing loss, because children and those with other types of hearing loss may require different amplification characteristics.
- Non-peer-reviewed articles will not be included (i.e. conference abstracts and theses).

INTRODUCTION

Hearing loss is the most prevalent sensory deficit, affecting more than one-fifth of the world's population[1, 2]. Untreated hearing loss reduces peoples' ability to communicate effectively, which could lead to social isolation, depression and decreased quality of life[3]. Hearing loss is associated with an increased risk of cognitive decline and dementia but causality is unknown[4]. The rate of unemployment among individuals with hearing loss is higher than that of the general population, costing the UK economy about £25 billion annually in terms of lost economic output[5].

The most prevalent type of hearing loss in adults is sensorineural loss, accounting for more than 90% of all adults with hearing loss in the UK[6]. The primary intervention for permanent hearing loss is the use of acoustic hearing aids[7]. These devices are designed to restore audibility of low-level sounds, maximise intelligibility of conversational level sounds, and maintain comfortability for loud sounds[8]. Hearing aids reduce the handicap caused by mild and moderate hearing loss (e.g. decreased quality of life)[9].

In the UK, hearing aids are fitted and prescribed by state-registered hearing health professionals. The health care professional's decision is based on a combination of audiometric and non-audiometric factors (e.g., hearing threshold levels, type of hearing loss,

For peer review only

physical condition and patient preference, respectively). The optimal amplification characteristics for each hearing aid user are specified according to validated prescription formulae (e.g. National Acoustic Laboratories Non-Linear 2)[10], which takes into account the patient-specific data (e.g. gender, language and type and degree of hearing loss) to personalise hearing aid output. Modern hearing aid software can approximate the amplification characteristics and the use of this approach for hearing aid fitting is known as initial-fit. A real-ear measurement (REM), which is a tool that can be used to accurately measure the output of a hearing aid when it is coupled to the individual's ear via a soft probe-tube microphone[8], can also be used to guide the adjustment of the output of the hearing aid so that it matches the proposed amplification target that was provided by the validated prescription formula.

Numerous studies have shown that the amplification characteristics approximated by the hearing aid software are inaccurate and significantly deviate from prescription targets[11-13]. These studies also suggest that the use of REMs can improve matches with prescription targets. Given the available evidence, using REMs, to verify the output of hearing aids and achieve better matches with prescription targets, has been endorsed by the British Society of Audiology[14] and most international hearing organisations (e.g. American Speech-Language-Hearing Association)[15]. Nevertheless, it remains unclear if improved matching to prescription targets result in better outcomes.

Using REMs to match hearing aid output to prescription targets requires valuable clinical time, which could otherwise be used to deliver alternative health and hearing services (e.g., identifying and addressing other hearing problems).

Rational

There is no published systematic review examining the evidence on whether the use of REM improves patient-reported outcomes in adult hearing aid users. Determining the effectiveness of hearing aids fitted using REM would help decision-makers and stakeholders either to continue recommending or to abandon routine use of REM during adult hearing aid fittings. This is an urgent knowledge gap for both stakeholders and clinicians, given that the cost of the REM system is relatively high and it consumes a large amount of clinicians' valuable time. The findings have implications for the emerging category of over-the-counter or direct-

For peer review only

to-consumer hearing aids. If fitting hearing aids using REM does not benefit patients, then one of the potential obstacles to people fitting their own hearing aids is overcome.

Objectives

The objective of this review is to systematically evaluate the current evidence on whether the use of REMs to match the hearing aid's output to one of the validated prescription targets improves outcomes in adult hearing aid users.

METHOD AND ANALYSIS

The protocol of this systematic review has been pre-registered with the International Prospective Register of Systematic Reviews (PROSPERO; CRD42020166074). The systematic review's method will be reported in accordance with Preferred Reporting Items for Systematic review and Meta-Analyses (PRISMA-P) guidelines[16].

Eligibility criteria

The inclusion and exclusion criteria for studies are reported in accordance with participants, interventions, comparators, outcomes and study designs (PICOS) elements.

Participants

Participants will include adults (≥ 18 years old) with any specified degree of sensorineural or mixed hearing loss. Studies that report only a qualitative description of age and threshold of hearing will also be included. If both children and adults are included in a clinical trial, the study will be excluded unless the data were interpreted and reported independently. Participants with other types of hearing loss (i.e. conductive or fluctuating hearing loss) will be excluded.

Interventions

The intervention that will be included is conventional acoustic hearing aids that were programmed to a prescription target using a real-ear measurement system. Assistive listening devices, hearables personal sound amplification products, and direct-to-consumer hearing devices will be excluded. Implantable devices (e.g. cochlear implants), bone conduction hearing aids or contralateral routing of sounds hearing aids will also be excluded.

For peer review only

Comparators

The comparisons of interest are hearing aids that are programmed to the manufacturers' approximation of the wearers hearing loss without REMs (i.e. initial fit approach).

Outcomes

The outcomes of interest are hearing-specific health-related quality of life (e.g. Hearing Handicap Inventory for the Elderly)[17], self-reported listening ability (e.g. Abbreviated Profile of Hearing Aid Benefit)[18], composite self-report measures (e.g. International Outcomes Inventory for Hearing Aids)[19], speech recognition in quiet or noisy environments, generic health-related quality of life, hours of use per day, number of required follow-up care sessions (i.e. for further fine-tuning) and adverse events (e.g. noise-induced hearing loss).

Study designs

Randomised controlled trials and non-randomised controlled trials will be included. Case reports, conference abstracts, book chapters, dissertations, theses, reviews and clinical guidelines will be excluded.

Information sources

Studies that meet the aforementioned eligibility criteria will be identified using a systematic search strategy of the following databases: MEDLINE, EMBASE, PsycINFO, CINAHL, Web of Science and CENTRAL via Cochrane Library. No search restrictions will be applied in terms of the publication's language, status and year.

The reference lists of the included publications will be manually scanned to identify further studies. Using Google Scholar "cited by" feature, publications that have cited any of the included studies will be screened to identify additional relevant articles. Prior to analysis, the searches will be repeated to identify any other relevant studies if there is a significant delay between searches and a manuscript's acceptance for publication (e.g. > 6 months).

Search strategy

The search protocol and methods will be developed by a medical information specialist from Systematic Review Solutions Limited. The included search terms will be based on experts'

For peer review only

opinion, free text and controlled terms from Medical Subject Headings (MeSH), Excerpta Medica Tree (EMTREE) and CINAHL headings. The search terms will include many free text terms because truncations may not work well with phrases (e.g. Hearing difficult* might not capture Hearing difficulties). The search strategies for all databases are reported in Appendix 1.

Study records

Data management (IA, KJM and HD)

IA will export the title and abstract of all of the identified articles to an Excel spreadsheet so that they can be easily screened against the eligibility criteria. The reason for excluding any article can also be documented and assessed by KJM and HD. Each article will be assigned a unique number that is linked to the full details of the article.

Selection process (IA, KJM and HD)

Search results, including titles, authors' details, publication years, publication journals and abstracts, will be extracted to Endnote X9 Reference Management software (Clarivate Analytics, 2018) or Microsoft Excel (2016). The same software will also be used to remove any duplications prior to the initial screening. The title and abstract of all identified studies will be screened independently by IA and KJM to determine eligibility for inclusion. A further inspection will be used when there is a discrepancy between the two investigators; this will include assessing the full article. Any disagreement will be resolved by discussion and/or by consulting with the third author (HD). Full-text will be retained for all articles that match the inclusion criteria. Following PRISMA- recommendations [20], a flow diagram will be used to present the study selection process.

Data collection process and data items (IA, KJM and HD)

IA and KJM will independently extract the data from the included studies. Should any discrepancies arise, these will be resolved through discussion or consultation with the third author (HD). The data will be extracted in a pre-designed data extraction form adapted from the Cochrane Handbook[21]. The extracted data will include but will not be limited to authors (year), methods, participants, intervention and outcomes (see Table 1). Data presented on

For peer review only

graphical forms will be extracted using an online extraction tool (e.g., WebPlotDigitizer; https://automeris.io/WebPlotDigitizer) when necessary.

Table 1 Data items

General information	Authors (year)
	Title
Method	Study design
	Total study duration
	Sequence generation
	Sequence concealment
	Blinding
Participants	Total number
	Country / Setting
	Age
	Sex
	Inclusion and exclusion criteria
Intervention	Intervention group
	Comparator group
Outcome	Primary outcome
	Secondary outcome(s)
Funding source	
Declaration of interest	
Notes	

Risk of bias individual studies (IA, KJM and HD)

The assessment of the risk of bias will be conducted independently by IA and KJM. Disagreements will be resolved through discussion or consulting with the third author (HD). Given the limited number of randomised controlled trials in the field of audiology, it is anticipated that most of the extracted studies will be non-randomised controlled trials; therefore, the Downs and Black[22] checklist will be used because it is easy to administer, has a well-established validity and reliability and can be used to assess the methodological quality of both randomised and non-randomised studies. Because knowledge on the clinically important differences in the outcomes of hearing aids is lacking, the scoring for the final item

For peer review only

(i.e. number 27) will be modified so that one point will be awarded if power calculation was conducted and zero points will be awarded if was not. Consequently, the maximum score will be 28. Articles scoring 26–28, 20–25, 15–19 and < 14 will be regarded as having excellent, good, fair and poor quality, respectively. The assessment of the risk of bias will be conducted by the primary and secondary authors independently.

Data synthesis

A meta-analysis will be conducted if enough data are available (i.e. more than one study). If the included studies used similar continuous outcome measures, the mean differences will be calculated with the 95% confidence interval (CI). When different outcome measures are used, the standardised mean difference will be measured along with 95% CI. If the included studies used similar dichotomous outcome measures, the risk ratio will be calculated. If the statistical heterogeneity is low, fixed-effect meta-analyses will be computed; otherwise, only the random-effect meta-analysis will be calculated. For each meta-analysis, the effect size estimate will be calculated using the generic inverse of variance. The effect estimate will be reported along with its 95% CI. Forest plots will be used to present these results. Asymmetrical distribution of continuous outcomes (i.e. skewed data) will be assessed by subtracting the lowest possible value from the mean and then dividing it by the SD. A ratio below 2 or 1 either suggests or indicates a skewed distribution, respectively[23]. Skewed data will be log-transformed where possible to reduce the skew distribution.

If meta-analysis is not appropriate, the risk of bias of each individual study will be assessed and the findings will be systematically reported

Assessment of reporting bias

The existence of publication bias will be visually inspected using a funnel plot of the study size or precision as a function of intervention effect estimates.

Assessment of heterogeneity

The percentage of variability between studies' outcomes that is due to heterogeneity rather than random error will be computed using an I^2 statistic. Given that the absolute threshold of I^2 is meaningless, the results will be interpreted as either low (0–40%), medium (41–60%) or high (61–100%) heterogeneity.

For peer review only

Dealing with missing data

The corresponding authors will be contacted, where necessary, if any of the data are missing. Data that are only presented in graphical forms will be extracted using the detailed method explained in the section titled Data collection process and data items. In case the standard deviations (SDs) are missing and cannot be obtained from the corresponding authors, they will be estimated from the available data (e.g. 95% CI and standard errors). The reasons for the missing data will be assessed to identify whether they are missing at random or not.

Subgroup analysis

Where heterogeneity is identified, subgroup analysis will be performed to determine whether age (i.e. ≤ 55 or > 55 years), severity of hearing loss and experience with hearing aids (i.e. first-time or experienced users) are the possible sources of heterogeneity.

Confidence in cumulative estimate (IA, KJM and HD)

The quality of each outcome measure will be rated as high, moderate, low or very low using a well-developed assessment tool, the Grading of Recommendations, Assessment, Development and Evaluations tool (GRADE)[24]. The quality rating assigned to each outcome measure reflects our level of confidence as to the veracity of the drawn estimate effect. The GRADE tool takes into account five principal domains: study limitations (e.g. blinding and allocation concealment), inconsistency (e.g., variabilities in effect size), indirectness (e.g. differences in population), and imprecision (e.g. broad CI) and publication bias (e.g. selective reporting of positive outcomes). Randomised controlled trials without serious shortcomings will be rated as high-quality evidence. That is, our confidence level is high enough to conclude that the true effect is close to the drawn estimate effect. Conversely, non-randomised control trials (e.g. observational studies) will be assigned a low rating. In other words, the estimated effect may vary considerably from the true estimate of the effect. However, the assigned rating to randomised and non-randomised controlled trials could be subject to either upgrading or downgrading by either one or two points on the basis of the seriousness of the above-mentioned assessment domains. A thorough discussion of these factors can be found in Schünemann et al (2013)[25]. IA and KJM will carry out the assessment independently and disagreements will be resolved by discussion and/or by consulting HD.

For peer review only

ETHICS AND DISSEMINATION

Ethical approval will not be sought because this systematic review will only retrieve and analyse data from published studies. Review results will be published in a peer-reviewed journal, presented at relevant scientific conferences and disseminated on social media.

Contributor IA is the guarantor of this review. IA developed and prepared the review protocol. The search procedure has been developed by a medical information specialist from Systematic Review Solutions Limited. KJM and HD contributed to the development of inclusion and exclusion criteria, search strategy and assessment of the risk of bias. KJM and HD also critically reviewed and provided feedback on earlier versions of the protocol. This manuscript has been approved by all contributors.

Funding NIHR Manchester Biomedical Research Centre (funder reference: IS-BRC-1215-20007)

Competing interest None declared.

ACKNOWLEDGEMENTS

IA, KJM and HD are supported by the NIHR Manchester Biomedical research Centre. The first author is also supported by the Deanship of Scientific Research at the College of Applied Medical Sciences Research Center at King Saud University.

For peer review only

Reference:

1. Mathers C, Smith A, Concha M. Global burden of hearing loss in the year 2000. *Global Burden of Disease* 2000;18:1-30.
2. World Health Organization. Prevention of blindness and deafness: World Health Organization; 2018 [Available from: https://www.who.int/pbd/deafness/estimates/en/ accessed 06/10/2019 2019.
3. Davis A, Smith P, Ferguson M, et al. Acceptability, benefit and costs of early screening for hearing disability: a study of potential screening tests and models. *Health Technol Assess* 2007;11(42):1-294. [published Online First: 2007/10/12]
4. Loughrey DG, Kelly ME, Kelley GA, et al. Association of Age-Related Hearing Loss With Cognitive Function, Cognitive Impairment, and Dementia: A Systematic Review and Meta-analysis. *JAMA Otolaryngol Head Neck Surg* 2018;144(2):115-26. doi: 10.1001/jamaoto.2017.2513 [published Online First: 2017/12/10]
5. International Longevity Centre UK. Commission on Hearing Loss: Final Report, 2014.
6. Action on Hearing Loss. Hearing Matters. UK, 2015.
7. Kochkin S. MarkeTrak VIII: 25-year trends in the hearing health market. *Hearing Review* 2009;16(11):12-31.
8. Dillon H. Hearing Aids: Thieme 2012.
9. Ferguson MA, Kitterick PT, Chong LY, et al. Hearing aids for mild to moderate hearing loss in adults. *Cochrane Db Syst Rev* 2017(9) doi: ARTN CD012023

10.1002/14651858.CD012023.pub2
10. Keidser G, Dillon H, Flax M, et al. The NAL-NL2 Prescription Procedure. *Audiol Res* 2011;1(1):e24. doi: 10.4081/audiores.2011.e24 [published Online First: 2011/05/10]
11. Munro KJ, Puri R, Bird J, et al. Using probe-microphone measurements to improve the match to target gain and frequency response slope, as a function of earmould style, frequency, and input level. *Int J Audiol* 2016;55(4):215-23. doi: 10.3109/14992027.2015.1104736 [published Online First: 2015/11/07]
12. Aazh H, Moore BC. The value of routine real ear measurement of the gain of digital hearing aids. *J Am Acad Audiol* 2007;18(8):653-64. doi: 10.3766/jaaa.18.8.3 [published Online First: 2008/03/11]
13. Aarts NL, Caffee CS. Manufacturer predicted and measured REAR values in adult hearing aid fitting: accuracy and clinical usefulness. *Int J Audiol* 2005;44(5):293-301. doi: 10.1080/14992020500057830 [published Online First: 2005/07/21]
14. British Society of Audiology. Guidance on the verification of hearing devices using probe microphone measurements. Seafield, England: British Society of Audiology, 2018:1 - 32.
15. Valente M, Bentler R, Kaplan HS, et al. Guidelines for hearing aid fitting for adults. . *American Journal of Audiology* 1998;7(1):5 - 13. doi: 10.1044/1059-0889.0701.05.
16. Shamseer L, Moher D, Clarke M, et al. Preferred reporting items for systematic review and meta-analysis protocols (PRISMA-P) 2015: elaboration and explanation. *Bmj* 2015;350:g7647. doi: 10.1136/bmj.g7647 [published Online First: 2015/01/04]
17. Humes LE, Wilson DL, Barlow NN, et al. Changes in hearing-aid benefit following 1 or 2 years of hearing-aid use by older adults. *J Speech Lang Hear Res* 2002;45(4):772-82. doi: 10.1044/1092-4388(2002/062) [published Online First: 2002/08/30]
18. Cox RMA, G. C. The abbreviated profile of hearing aid benefit. *Ear Hear* 1995;16(2):176-86. doi: 10.1097/00003446-199504000-00005 [published Online First: 1995/04/01]
19. Cox RM, Alexander GC. The International Outcome Inventory for Hearing Aids (IOI-HA): psychometric properties of the English version. *Int J Audiol* 2002;41(1):30-5. doi: 10.3109/14992020209101309 [published Online First: 2002/12/07]

For peer review only

20. Moher D, Liberati A, Tetzlaff J, et al. Preferred reporting items for systematic reviews and meta-analyses: the PRISMA statement. *PLoS medicine* 2009;6(7):e1000097. doi: 10.1371/journal.pmed.1000097 [published Online First: 2009/07/22]
21. Higgins JPT, Green S. Cochrane handbook for systematic reviews of interventions. Chichester, England ; Hoboken, NJ: Wiley-Blackwell 2008.
22. Downs SH, Black N. The feasibility of creating a checklist for the assessment of the methodological quality both of randomised and non-randomised studies of health care interventions. *Journal of epidemiology and community health* 1998;52(6):377-84. doi: 10.1136/jech.52.6.377 [published Online First: 1998/10/09]
23. Deeks JJ, Higgins JP, Altman DG. Analysing data and undertaking meta-analyses. In: Higgins JPT, Thomas J, Chandler J, et al., eds. Cochrane Handbook for Systematic Reviews of Interventions. Hoboken: WILEY Blackwell 2019.
24. Atkins D, Best D, Briss PA, et al. Grading quality of evidence and strength of recommendations. *Bmj* 2004;328(7454):1490. doi: 10.1136/bmj.328.7454.1490 [published Online First: 2004/06/19]
25. Schünemann H, Brożek J, Guyatt G, et al. GRADE Handbook2013.

For peer review only

Appendix 1: Search Strategies

A. COCHRANE Library

(("Hearing Loss" OR "hearing loss" OR "hearing losses" OR deaf* OR "Hearing problem" OR "hearing problems" OR hypoacus* OR "hearing impaired" OR "hearing impairment" OR "hearing impairments" OR "hard of hearing" OR "hearing difficulties" OR "hearing difficulty" OR "hearing disabilities" OR "hearing disability" OR "Hearing Disorders" OR "hearing disorder" OR "hearing disorders" OR "hearing loss*" OR "Hearing problem*" OR "hearing impair*" OR "hearing difficult*" OR "hearing disab*" OR "hearing disorder*") AND ("Hearing Aids" OR "hearing aid" OR "hearing aids" OR "hearing device" OR "hearing devices" OR "acoustic aid" OR "acoustic aids" OR "ear molds" OR "ear mold" OR "HA" OR "aid") AND ("real ear measurement" OR "real ear measurements" OR "real ear measure" OR "real ear measures" OR "real-ear measurement" OR "real-ear measurements" OR "real-ear measure" OR "real-ear measures" OR "REM" OR PMM* OR "probe microphone" OR "probe microphones" OR "probe mic" OR "probe mics" OR "probe microphone measurement" OR "probe microphone measurements" OR "probe microphone measure" OR "probe microphone measures" OR "probe-microphone" OR "probe-microphones" OR "probe-microphone measurement" OR "probe-microphone measurements" OR "probe-microphone measure" OR "probe-microphone measures" OR "initial fit" OR "initial fits" OR "initial fitting" OR "initial fittings" OR "real ear" OR "real ears" OR "real-ear" OR "real-ears" OR "hearing aid fit" OR "hearing aid fitter" OR "hearing aid fitting" OR "hearing aid fittings")):ti,ab,kw NOT (("Child" OR "Infant") NOT "Adult*"):ti

B. Embase

(((exp *"Hearing Aid"/ OR "hearing aid".ti OR "hearing aids".ti OR "hearing device".ti OR "hearing devices".ti OR "acoustic aid".ti OR "acoustic aids".ti OR "ear molds".ti OR "ear mold".ti OR "HA".ti OR "aid".ti) AND ("real ear measurement".ti OR "real ear measurements".ti OR "real ear measure".ti OR "real ear measures".ti OR "real-ear measurement".ti OR "real-ear measurements".ti OR "real-ear measure".ti OR "real-ear measures".ti OR "REM".ti OR PMM*.ti OR "probe microphone".ti OR "probe microphones".ti OR "probe mic".ti OR "probe mics".ti OR "probe microphone measurement".ti OR "probe microphone measurements".ti OR "probe microphone measure".ti OR "probe microphone measures".ti OR "probe-microphone".ti OR "probe-microphones".ti OR "probe-microphone measurement".ti OR "probe-microphone measurements".ti OR "probe-microphone measure".ti OR "probe-microphone measures".ti OR "initial fit".ti OR "initial fits".ti OR "initial fitting".ti OR "initial fittings".ti OR "real ear".ti OR "real ears".ti OR "real-ear".ti OR "real-ears".ti OR "hearing aid fit".ti OR "hearing aid fitter".ti OR "hearing aid fitting".ti OR "hearing aid fittings".ti) NOT (exp "juvenile"/ NOT exp "adult"/) NOT (conference review or conference abstract).pt NOT ("case report"/ OR ((exp "review"/ OR exp "Practice Guideline"/ OR "guideline".ti OR "guidelines".ti) NOT exp "Clinical Study"/))) OR ((exp *"Hearing Aid"/ OR "hearing aid".ti,ab OR "hearing

aids".ti,ab OR "hearing device".ti,ab OR "hearing devices".ti,ab OR "acoustic aid".ti,ab OR "acoustic aids".ti,ab OR "ear molds".ti,ab OR "ear mold".ti,ab OR "HA".ti,ab OR "aid".ti,ab) AND ("real ear measurement".mp OR "real ear measurements".mp OR "real ear measure".mp OR "real ear measures".mp OR "real-ear measurement".mp OR "real-ear measurements".mp OR "real-ear measure".mp OR "real-ear measures".mp OR "REM".mp OR PMM*.mp OR "probe microphone".mp OR "probe microphones".mp OR "probe mic".mp OR "probe mics".mp OR "probe microphone measurement".mp OR "probe microphone measurements".mp OR "probe microphone measure".mp OR "probe microphone measures".mp OR "probe-microphone".mp OR "probe-microphones".mp OR "probe-microphone measurement".mp OR "probe-microphone measurements".mp OR "probe-microphone measure".mp OR "probe-microphone measures".mp OR "initial fit".mp OR "initial fits".mp OR "initial fitting".mp OR "initial fittings".mp OR "real ear".mp OR "real ears".mp OR "real-ear".mp OR "real-ears".mp OR "hearing aid fit".mp OR "hearing aid fitter".mp OR "hearing aid fitting".mp OR "hearing aid fittings".mp) AND (exp *"Hearing Impairment"/ OR "hearing loss".ti,ab OR "hearing losses".ti,ab OR deaf*.ti,ab OR "Hearing problem".ti,ab OR "hearing problems".ti,ab OR hypoacus*.ti,ab OR "hearing impaired".ti,ab OR "hearing impairment".ti,ab OR "hearing impairments".ti,ab OR "hard of hearing".ti,ab OR "hearing difficulties".ti,ab OR "hearing difficulty".ti,ab OR "hearing disabilities".ti,ab OR "hearing disability".ti,ab OR "hearing loss*".ti,ab OR "Hearing problem*".ti,ab OR "hearing impair*".ti,ab OR "hearing difficult*".ti,ab OR "hearing disab*".ti,ab OR "hearing disorder*".ti,ab) AND ("randomised".mp OR "randomized".mp OR "nonrandomised".mp OR "nonrandomised".mp OR exp "Randomization"/ OR "Randomized Controlled Trial"/ OR exp "Controlled Clinical Trial"/ OR "Equivalence Trial"/ OR "Pragmatic Trial"/ OR "quasi experimental study"/ OR "Random Allocation".mp OR "Controlled Clinical Trial".mp OR "Controlled Clinical Trials".mp OR "Controlled Trial".mp OR "Controlled Trials".mp OR "Equivalence Trial".mp OR "Pragmatic Clinical Trial".mp OR "Equivalence Trials".mp OR "Pragmatic Clinical Trials".mp OR "RCT".mp OR "RCTs".mp OR "Quasi-Experimental".mp OR "Quasi-Experiment*".mp OR "QuasiExperimental".mp OR QuasiExperiment*.mp OR "Crossover Procedure"/ OR "Cross-Over".mp OR "CrossOver".mp OR "retrospective".mp OR Retrospectiv*.mp OR "Retrospective Study"/ OR "Prospective Study"/ OR "prospective".mp OR prospectiv*.mp OR exp "Evaluation Study"/ OR "evaluate".mp OR evaluat*.mp OR exp "Comparative Study"/ OR "compare".mp OR "comparative".mp OR compar*.mp) NOT (exp "juvenile"/ NOT exp "adult"/) NOT (conference review or conference abstract).pt NOT ("case report"/ OR ((exp "review"/ OR exp "Practice Guideline"/ OR "guideline".ti OR "guidelines".ti) NOT exp "Clinical Study"/))))

C. Emcare

(((exp *"Hearing Aid"/ OR "hearing aid".ti OR "hearing aids".ti OR "hearing device".ti OR "hearing devices".ti OR "acoustic aid".ti OR "acoustic aids".ti OR "ear molds".ti OR "ear mold".ti OR "HA".ti OR "aid".ti) AND ("real ear measurement".ti OR "real ear measurements".ti OR "real ear measure".ti OR "real ear measures".ti OR "real-ear measurement".ti OR "real-ear measurements".ti OR "real-ear measure".ti OR "real-ear

measures".ti OR "REM".ti OR PMM*.ti OR "probe microphone".ti OR "probe microphones".ti OR "probe mic".ti OR "probe mics".ti OR "probe microphone measurement".ti OR "probe microphone measurements".ti OR "probe microphone measure".ti OR "probe microphone measures".ti OR "probe-microphone".ti OR "probe-microphones".ti OR "probe-microphone measurement".ti OR "probe-microphone measurements".ti OR "probe-microphone measure".ti OR "probe-microphone measures".ti OR "initial fit".ti OR "initial fits".ti OR "initial fitting".ti OR "initial fittings".ti OR "real ear".ti OR "real ears".ti OR "real-ear".ti OR "real-ears".ti OR "hearing aid fit".ti OR "hearing aid fitter".ti OR "hearing aid fitting".ti OR "hearing aid fittings".ti) NOT (exp "juvenile"/ NOT exp "adult"/) NOT (conference review or conference abstract).pt NOT ("case report"/ OR ((exp "review"/ OR exp "Practice Guideline"/ OR "guideline".ti OR "guidelines".ti) NOT exp "Clinical Study"/))) OR ((exp *"Hearing Aid"/ OR "hearing aid".ti,ab OR "hearing aids".ti,ab OR "hearing device".ti,ab OR "hearing devices".ti,ab OR "acoustic aid".ti,ab OR "acoustic aids".ti,ab OR "ear molds".ti,ab OR "ear mold".ti,ab OR "HA".ti,ab OR "aid".ti,ab) AND ("real ear measurement".mp OR "real ear measurements".mp OR "real ear measure".mp OR "real ear measures".mp OR "real-ear measurement".mp OR "real-ear measurements".mp OR "real-ear measure".mp OR "real-ear measures".mp OR "REM".mp OR PMM*.mp OR "probe microphone".mp OR "probe microphones".mp OR "probe mic".mp OR "probe mics".mp OR "probe microphone measurement".mp OR "probe microphone measurements".mp OR "probe microphone measure".mp OR "probe microphone measures".mp OR "probe-microphone".mp OR "probe-microphones".mp OR "probe-microphone measurement".mp OR "probe-microphone measurements".mp OR "probe-microphone measure".mp OR "probe-microphone measures".mp OR "initial fit".mp OR "initial fits".mp OR "initial fitting".mp OR "initial fittings".mp OR "real ear".mp OR "real ears".mp OR "real-ear".mp OR "real-ears".mp OR "hearing aid fit".mp OR "hearing aid fitter".mp OR "hearing aid fitting".mp OR "hearing aid fittings".mp) AND (exp *"Hearing Impairment"/ OR "hearing loss".ti,ab OR "hearing losses".ti,ab OR deaf*.ti,ab OR "Hearing problem".ti,ab OR "hearing problems".ti,ab OR hypoacus*.ti,ab OR "hearing impaired".ti,ab OR "hearing impairment".ti,ab OR "hearing impairments".ti,ab OR "hard of hearing".ti,ab OR "hearing difficulties".ti,ab OR "hearing difficulty".ti,ab OR "hearing disabilities".ti,ab OR "hearing disability".ti,ab OR "hearing loss*".ti,ab OR "Hearing problem*".ti,ab OR "hearing impair*".ti,ab OR "hearing difficult*".ti,ab OR "hearing disab*".ti,ab OR "hearing disorder*".ti,ab) AND ("randomised".mp OR "randomized".mp OR "nonrandomised".mp OR "nonrandomised".mp OR exp "Randomization"/ OR "Randomized Controlled Trial"/ OR exp "Controlled Clinical Trial"/ OR "Equivalence Trial"/ OR "Pragmatic Trial"/ OR "quasi experimental study"/ OR "Random Allocation".mp OR "Controlled Clinical Trial".mp OR "Controlled Clinical Trials".mp OR "Controlled Trial".mp OR "Controlled Trials".mp OR "Equivalence Trial".mp OR "Pragmatic Clinical Trial".mp OR "Equivalence Trials".mp OR "Pragmatic Clinical Trials".mp OR "RCT".mp OR "RCTs".mp OR "Quasi-Experimental".mp OR "Quasi-Experiment*".mp OR "QuasiExperimental".mp OR QuasiExperiment*.mp OR "Crossover Procedure"/ OR "Cross-Over".mp OR "CrossOver".mp OR "retrospective".mp OR Retrospectiv*.mp OR "Retrospective Study"/ OR "Prospective Study"/ OR "prospective".mp OR prospectiv*.mp OR exp "Evaluation Study"/ OR "evaluate".mp OR evaluat*.mp OR exp "Comparative Study"/ OR "compare".mp OR "comparative".mp OR

compar*.mp) NOT (exp "juvenile"/ NOT exp "adult"/) NOT (conference review or conference abstract).pt NOT ("case report"/ OR ((exp "review"/ OR exp "Practice Guideline"/ OR "guideline".ti OR "guidelines".ti) NOT exp "Clinical Study"/))))

D. MEDLINE

(("Hearing Loss"/ OR "hearing loss".mp OR "hearing losses".mp OR deaf*.mp OR "Hearing problem".mp OR "hearing problems".mp OR hypoacus*.mp OR "hearing impaired".mp OR "hearing impairment".mp OR "hearing impairments".mp OR "hard of hearing".mp OR "hearing difficulties".mp OR "hearing difficulty".mp OR "hearing disabilities".mp OR "hearing disability".mp OR exp "Hearing Disorders"/ OR "hearing disorder".mp OR "hearing disorders".mp OR hearing loss*.mp OR Hearing problem*.mp OR hearing impair*.mp OR hearing difficult*.mp OR hearing disab*.mp OR hearing disorder*.mp) AND (exp "Hearing Aids"/ OR "hearing aid".mp OR "hearing aids".mp OR "hearing device".mp OR "hearing devices".mp OR "acoustic aid".mp OR "acoustic aids".mp OR "ear molds".mp OR "ear mold".mp OR "HA".ti,ab OR "aid".mp) AND ("real ear measurement".mp OR "real ear measurements".mp OR "real ear measure".mp OR "real ear measures".mp OR "real-ear measurement".mp OR "real-ear measurements".mp OR "real-ear measure".mp OR "real-ear measures".mp OR "REM".mp OR PMM*.mp OR "probe microphone".mp OR "probe microphones".mp OR "probe mic".mp OR "probe mics".mp OR "probe microphone measurement".mp OR "probe microphone measurements".mp OR "probe microphone measure".mp OR "probe microphone measures".mp OR "probe-microphone".mp OR "probe-microphones".mp OR "probe-microphone measurement".mp OR "probe-microphone measurements".mp OR "probe-microphone measure".mp OR "probe-microphone measures".mp OR "initial fit".mp OR "initial fits".mp OR "initial fitting".mp OR "initial fittings".mp OR "real ear".mp OR "real ears".mp OR "real-ear".mp OR "real-ears".mp OR "hearing aid fit".mp OR "hearing aid fitter".mp OR "hearing aid fitting".mp OR "hearing aid fittings".mp) AND ("randomised".mp OR "randomized".mp OR "nonrandomised".mp OR "nonrandomised".mp OR "Random Allocation"/ OR exp "Randomized Controlled Trial"/ OR exp "Randomized Controlled Trials as Topic"/ OR "Non-Randomized Controlled Trials as Topic"/ OR exp "Controlled Clinical Trial"/ OR exp "Equivalence Trial"/ OR exp "Pragmatic Clinical Trial"/ OR exp "Equivalence Trials as Topic"/ OR exp "Pragmatic Clinical Trials as Topic"/ OR "Random Allocation".mp OR "Controlled Clinical Trial".mp OR "Controlled Clinical Trials".mp OR "Controlled Trial".mp OR "Controlled Trials".mp OR "Equivalence Trial".mp OR "Pragmatic Clinical Trial".mp OR "Equivalence Trials".mp OR "Pragmatic Clinical Trials".mp OR "RCT".mp OR "RCTs".mp OR "Quasi-Experimental".mp OR Quasi-Experiment*.mp OR "QuasiExperimental".mp OR QuasiExperiment*.mp OR "Cross-Over Studies"/ OR "Cross-Over".mp OR "CrossOver".mp OR "retrospective".mp OR Retrospectiv*.mp OR exp "Retrospective Studies"/ OR "Prospective Studies"/ OR "prospective".mp OR prospectiv*.mp OR exp "Evaluation Study"/ OR exp "Evaluation Studies as Topic"/ OR "evaluate".mp OR evaluat*.mp OR exp "Comparative Study"/ OR "compare".mp OR "comparative".mp OR compar*.mp) NOT ((exp "Child"/ OR exp "Infant"/) NOT exp "Adult"/) NOT (("Case Reports"/ OR "case report".ti OR "Review"/ OR

For peer review only

"review".ti OR exp "Guideline"/ OR "guideline".ti OR "guidelines".ti) NOT (exp "Clinical Study"/ OR "trial".ti OR "RCT".ti)))

E. PsycINFO

(TX("Hearing Loss" OR "hearing loss" OR "hearing losses" OR deaf* OR "Hearing problem" OR "hearing problems" OR hypoacus* OR "hearing impaired" OR "hearing impairment" OR "hearing impairments" OR "hard of hearing" OR "hearing difficulties" OR "hearing difficulty" OR "hearing disabilities" OR "hearing disability" OR "Hearing Disorders" OR "hearing disorder" OR "hearing disorders" OR "hearing loss*" OR "Hearing problem*" OR "hearing impair*" OR "hearing difficult*" OR "hearing disab*" OR "hearing disorder*") AND TX("Hearing Aids" OR "hearing aid" OR "hearing aids" OR "hearing device" OR "hearing devices" OR "acoustic aid" OR "acoustic aids" OR "ear molds" OR "ear mold" OR "HA" OR "aid") AND TX("real ear measurement" OR "real ear measurements" OR "real ear measure" OR "real ear measures" OR "real-ear measurement" OR "real-ear measurements" OR "real-ear measure" OR "real-ear measures" OR "REM" OR PMM* OR "probe microphone" OR "probe microphones" OR "probe mic" OR "probe mics" OR "probe microphone measurement" OR "probe microphone measurements" OR "probe microphone measure" OR "probe microphone measures" OR "probe-microphone" OR "probe-microphones" OR "probe-microphone measurement" OR "probe-microphone measurements" OR "probe-microphone measure" OR "probe-microphone measures" OR "initial fit" OR "initial fits" OR "initial fitting" OR "initial fittings" OR "real ear" OR "real ears" OR "real-ear" OR "real-ears" OR "hearing aid fit" OR "hearing aid fitter" OR "hearing aid fitting" OR "hearing aid fittings") AND TX("randomised" OR "randomized" OR "nonrandomised" OR "nonrandomised" OR "Random Allocation" OR "Randomized Controlled Trial" OR "Randomized Controlled Trials as Topic" OR "Non-Randomized Controlled Trials as Topic" OR "Controlled Clinical Trial" OR "Equivalence Trial" OR "Pragmatic Clinical Trial" OR "Equivalence Trials as Topic" OR "Pragmatic Clinical Trials as Topic" OR "Random Allocation" OR "Controlled Clinical Trial" OR "Controlled Clinical Trials" OR "Controlled Trial" OR "Controlled Trials" OR "Equivalence Trial" OR "Pragmatic Clinical Trial" OR "Equivalence Trials" OR "Pragmatic Clinical Trials" OR "RCT" OR "RCTs" OR "Quasi-Experimental" OR Quasi-Experiment* OR "QuasiExperimental" OR QuasiExperiment* OR "Cross-Over Studies" OR "Cross-Over" OR "CrossOver" OR "retrospective" OR Retrospectiv* OR "Retrospective Studies" OR "Prospective Studies" OR "prospective" OR prospectiv* OR "Evaluation Study" OR "Evaluation Studies as Topic" OR "evaluate" OR evaluat* OR "Comparative Study" OR "compare" OR "comparative" OR compar*) NOT TI(("Child" OR "Infant") NOT "Adult") NOT TI(("Case Reports" OR "case report" OR "Review" OR "review" OR "Guideline" OR "guideline" OR "guidelines") NOT ("Clinical Study" OR "trial" OR "RCT")))

F. PubMed

For peer review only

(("Hearing Loss"[Mesh] OR "hearing loss"[tw] OR "hearing losses"[tw] OR deaf*[tw] OR "Hearing problem"[tw] OR "hearing problems"[tw] OR hypoacus*[tw] OR "hearing impaired"[tw] OR "hearing impairment"[tw] OR "hearing impairments"[tw] OR "hard of hearing"[tw] OR "hearing difficulties"[tw] OR "hearing difficulty"[tw] OR "hearing disabilities"[tw] OR "hearing disability"[tw] OR "Hearing Disorders"[mesh] OR "hearing disorder"[tw] OR "hearing disorders"[tw] OR hearing loss*[tw] OR Hearing problem*[tw] OR hearing impair*[tw] OR hearing difficult*[tw] OR hearing disab*[tw] OR hearing disorder*[tw]) AND ("Hearing Aids"[Mesh] OR "hearing aid"[tw] OR "hearing aids"[tw] OR "hearing device"[tw] OR "hearing devices"[tw] OR "acoustic aid"[tw] OR "acoustic aids"[tw] OR "ear molds"[tw] OR "ear mold"[tw] OR "HA"[tiab] OR "aid"[tw]) AND ("real ear measurement"[tw] OR "real ear measurements"[tw] OR "real ear measure"[tw] OR "real ear measures"[tw] OR "real-ear measurement"[tw] OR "real-ear measurements"[tw] OR "real-ear measure"[tw] OR "real-ear measures"[tw] OR "REM"[tw] OR PMM*[tw] OR "probe microphone"[tw] OR "probe microphones"[tw] OR "probe mic"[tw] OR "probe mics"[tw] OR "probe microphone measurement"[tw] OR "probe microphone measurements"[tw] OR "probe microphone measure"[tw] OR "probe microphone measures"[tw] OR "probe-microphone"[tw] OR "probe-microphones"[tw] OR "probe-microphone measurement"[tw] OR "probe-microphone measurements"[tw] OR "probe-microphone measure"[tw] OR "probe-microphone measures"[tw] OR "initial fit"[tw] OR "initial fits"[tw] OR "initial fitting"[tw] OR "initial fittings"[tw] OR "real ear"[tw] OR "real ears"[tw] OR "real-ear"[tw] OR "real-ears"[tw] OR "hearing aid fit"[tw] OR "hearing aid fitter"[tw] OR "hearing aid fitting"[tw] OR "hearing aid fittings"[tw]) AND ("randomised"[tw] OR "randomized"[tw] OR "nonrandomised"[tw] OR "nonrandomised"[tw] OR "Random Allocation"[Mesh] OR "Randomized Controlled Trial"[Ptyp] OR "Randomized Controlled Trials as Topic"[Mesh] OR "Non-Randomized Controlled Trials as Topic"[Mesh] OR "Controlled Clinical Trial"[ptyp] OR "Equivalence Trial"[ptyp] OR "Pragmatic Clinical Trial"[ptyp] OR "Equivalence Trials as Topic"[Mesh] OR "Pragmatic Clinical Trials as Topic"[Mesh] OR "Random Allocation"[tw] OR "Controlled Clinical Trial"[tw] OR "Controlled Clinical Trials"[tw] OR "Controlled Trial"[tw] OR "Controlled Trials"[tw] OR "Equivalence Trial"[tw] OR "Pragmatic Clinical Trial"[tw] OR "Equivalence Trials"[tw] OR "Pragmatic Clinical Trials"[tw] OR "RCT"[tw] OR "RCTs"[tw] OR "Quasi-Experimental"[tw] OR Quasi-Experiment*[tw] OR "QuasiExperimental"[tw] OR QuasiExperiment*[tw] OR "Cross-Over Studies"[Mesh] OR "Cross-Over"[tw] OR "CrossOver"[tw] OR "retrospective"[tw] OR Retrospectiv*[tw] OR "Retrospective Studies"[Mesh] OR "Prospective Studies"[Mesh] OR "prospective"[tw] OR prospectiv*[tw] OR "Evaluation Study"[ptyp] OR "Evaluation Studies as Topic"[Mesh] OR "evaluate"[tw] OR evaluat*[tw] OR "Comparative Study"[ptyp] OR "compare"[tw] OR "comparative"[tw] OR compar*[tw]) NOT (("Child"[mesh] OR "Infant"[mesh]) NOT "Adult"[mesh]) NOT (("Case Reports"[ptyp] OR "case report"[ti] OR "Review"[ptyp] OR "review"[ti] OR "Guideline"[Publication Type] OR "guideline"[ti] OR "guidelines"[ti]) NOT ("Clinical Study"[ptyp] OR "trial"[ti] OR "RCT"[ti])))

G. Web of Science

For peer review only

(ts=("Hearing Loss" OR "hearing loss" OR "hearing losses" OR deaf* OR "Hearing problem" OR "hearing problems" OR hypoacus* OR "hearing impaired" OR "hearing impairment" OR "hearing impairments" OR "hard of hearing" OR "hearing difficulties" OR "hearing difficulty" OR "hearing disabilities" OR "hearing disability" OR "Hearing Disorders" OR "hearing disorder" OR "hearing disorders" OR "hearing loss*" OR "Hearing problem*" OR "hearing impair*" OR "hearing difficult*" OR "hearing disab*" OR "hearing disorder*") AND ts=("Hearing Aids" OR "hearing aid" OR "hearing aids" OR "hearing device" OR "hearing devices" OR "acoustic aid" OR "acoustic aids" OR "ear molds" OR "ear mold" OR "HA" OR "aid") AND ts=("real ear measurement" OR "real ear measurements" OR "real ear measure" OR "real ear measures" OR "real-ear measurement" OR "real-ear measurements" OR "real-ear measure" OR "real-ear measures" OR "REM" OR PMM* OR "probe microphone" OR "probe microphones" OR "probe mic" OR "probe mics" OR "probe microphone measurement" OR "probe microphone measurements" OR "probe microphone measure" OR "probe microphone measures" OR "probe-microphone" OR "probe-microphones" OR "probe-microphone measurement" OR "probe-microphone measurements" OR "probe-microphone measure" OR "probe-microphone measures" OR "initial fit" OR "initial fits" OR "initial fitting" OR "initial fittings" OR "real ear" OR "real ears" OR "real-ear" OR "real-ears" OR "hearing aid fit" OR "hearing aid fitter" OR "hearing aid fitting" OR "hearing aid fittings") AND ts=("randomised" OR "randomized" OR "nonrandomised" OR "nonrandomised" OR "Random Allocation" OR "Randomized Controlled Trial" OR "Randomized Controlled Trials as Topic" OR "Non-Randomized Controlled Trials as Topic" OR "Controlled Clinical Trial" OR "Equivalence Trial" OR "Pragmatic Clinical Trial" OR "Equivalence Trials as Topic" OR "Pragmatic Clinical Trials as Topic" OR "Random Allocation" OR "Controlled Clinical Trial" OR "Controlled Clinical Trials" OR "Controlled Trial" OR "Controlled Trials" OR "Equivalence Trial" OR "Pragmatic Clinical Trial" OR "Equivalence Trials" OR "Pragmatic Clinical Trials" OR "RCT" OR "RCTs" OR "Quasi-Experimental" OR Quasi-Experiment* OR "QuasiExperimental" OR QuasiExperiment* OR "Cross-Over Studies" OR "Cross-Over" OR "CrossOver" OR "retrospective" OR Retrospectiv* OR "Retrospective Studies" OR "Prospective Studies" OR "prospective" OR prospectiv* OR "Evaluation Study" OR "Evaluation Studies as Topic" OR "evaluate" OR evaluat* OR "Comparative Study" OR "compare" OR "comparative" OR compar*) NOT ti=(("Child" OR "Infant") NOT "Adult") NOT ti=(("Case Reports" OR "case report" OR "Review" OR "review" OR "Guideline" OR "guideline" OR "guidelines") NOT ("Clinical Study" OR "trial" OR "RCT")) NOT dt=(meeting abstract))

For peer review only

Appendix 2

Preferred reporting items for systematic review and meta-analysis protocols (PRISMA-P) 2015

PRISMA-P 2015 Checklist

Section/topic	Item No	Checklist item	Information reported Yes	Information reported No	Page number
**ADMINISTRATIVE INFORMATION**					
**Title**					
Identification	1a	Identify the report as a protocol of a systematic review	☒	☐	1
Update	1b	If the protocol is for an update of a previous systematic review, identify as such	☐	☒	Not an update
**Registration**	2	If registered, provide the name of the registry (e.g., PROSPERO) and registration number in the Abstract	☒	☐	2
**Authors**					
Contact	3a	Provide name, institutional affiliation, and e-mail address of all protocol authors; provide physical mailing address of corresponding author	☒	☐	1
Contributions	3b	Describe contributions of protocol authors and identify the guarantor of the review	☒	☐	11
**Amendments**	4	If the protocol represents an amendment of a previously completed or published protocol, identify as such and list changes; otherwise, state plan for documenting important protocol amendments	☐	☒	Not an amendment
**Support**					
Sources	5a	Indicate sources of financial or other support for the review	☒	☐	11
Sponsor	5b	Provide name for the review funder and/or sponsor	☒	☐	11
Role of sponsor/funder	5c	Describe roles of funder(s), sponsor(s), and/or institution(s), if any, in developing the protocol	☒	☐	11
**INTRODUCTION**					
**Rationale**	6	Describe the rationale for the review in the context of what is already known	☒	☐	4-5
**Objectives**	7	Provide an explicit statement of the question(s) the review will address with reference to participants, interventions, comparators, and outcomes (PICO)	☒	☐	5
**METHODS**					

For peer review only

**Eligibility criteria**	8	Specify the study characteristics (e.g., PICO, study design, setting, time frame) and report characteristics (e.g., years considered, language, publication status) to be used as criteria for eligibility for the review	☒	☐	6
**Information sources**	9	Describe all intended information sources (e.g., electronic databases, contact with study authors, trial registers, or other grey literature sources) with planned dates of coverage	☒	☐	6-7
**Search strategy**	10	Present draft of search strategy to be used for at least one electronic database, including planned limits, such that it could be repeated	☒	☐	Appendix 1
STUDY RECORDS					
Data management	11a	Describe the mechanism(s) that will be used to manage records and data throughout the review	☒	☐	7
Selection process	11b	State the process that will be used for selecting studies (e.g., two independent reviewers) through each phase of the review (i.e., screening, eligibility, and inclusion in meta-analysis)	☒	☐	7
Data collection process	11c	Describe planned method of extracting data from reports (e.g., piloting forms, done independently, in duplicate), any processes for obtaining and confirming data from investigators	☒	☐	7-8
**Data items**	12	List and define all variables for which data will be sought (e.g., PICO items, funding sources), any pre-planned data assumptions and simplifications	☒	☐	7-8
**Outcomes and prioritization**	13	List and define all outcomes for which data will be sought, including prioritization of main and additional outcomes, with rationale	☒	☐	6
**Risk of bias in individual studies**	14	Describe anticipated methods for assessing risk of bias of individual studies, including whether this will be done at the outcome or study level, or both; state how this information will be used in data synthesis	☒	☐	8-9
DATA					
**Synthesis**	15a	Describe criteria under which study data will be quantitatively synthesized	☒	☐	9
	15b	If data are appropriate for quantitative synthesis, describe planned summary measures, methods of handling data, and methods of combining data from studies, including any planned exploration of consistency (e.g., I^2, Kendall's tau)	☒	☐	9
	15c	Describe any proposed additional analyses (e.g., sensitivity or subgroup analyses, meta-regression)	☒	☐	10
	15d	If quantitative synthesis is not appropriate, describe the type of summary planned	☒	☐	9
**Meta-bias(es)**	16	Specify any planned assessment of meta-bias(es) (e.g., publication bias across studies, selective reporting within studies)	☒	☐	9
**Confidence in cumulative evidence**	17	Describe how the strength of the body of evidence will be assessed (e.g., GRADE)	☒	☐	10

For peer review only

BMJ Open

Does probe-tube verification of real-ear hearing aid amplification characteristics improve outcomes in adult hearing aid users? A protocol for a systematic review

Journal:	*BMJ Open*
Manuscript ID	bmjopen-2020-038113.R1
Article Type:	Protocol
Date Submitted by the Author:	13-May-2020
Complete List of Authors:	Almufarrij, Ibrahim; The University of Manchester, Manchester Centre for Audiology and Deafness, School of Health Sciences, University of Manchester and the Manchester Academic Health Science Centre; King Saud University, Department of Rehabilitation Sciences<br>Munro, Kevin; The University of Manchester, Manchester Centre for Audiology and Deafness, School of Health Sciences, University of Manchester and the Manchester Academic Health Science Centre; Manchester University NHS Foundation Trust, Manchester Academic Health Science Centre<br>Dillon, Harvey; The University of Manchester, Manchester Centre for Audiology and Deafness, School of Health Sciences, University of Manchester and the Manchester Academic Health Science Centre; Macquarie University
**Primary Subject Heading**:	Ear, nose and throat/otolaryngology
Secondary Subject Heading:	Ear, nose and throat/otolaryngology, Evidence based practice
Keywords:	Audiology < OTOLARYNGOLOGY, OTOLARYNGOLOGY, Neurotology < OTOLARYNGOLOGY

SCHOLARONE™
Manuscripts

I, the Submitting Author has the right to grant and does grant on behalf of all authors of the Work (as defined in the below author licence), an exclusive licence and/or a non-exclusive licence for contributions from authors who are: i) UK Crown employees; ii) where BMJ has agreed a CC-BY licence shall apply, and/or iii) in accordance with the terms applicable for US Federal Government officers or employees acting as part of their official duties; on a worldwide, perpetual, irrevocable, royalty-free basis to BMJ Publishing Group Ltd ("BMJ") its licensees and where the relevant Journal is co-owned by BMJ to the co-owners of the Journal, to publish the Work in this journal and any other BMJ products and to exploit all rights, as set out in our licence.

The Submitting Author accepts and understands that any supply made under these terms is made by BMJ to the Submitting Author unless you are acting as an employee on behalf of your employer or a postgraduate student of an affiliated institution which is paying any applicable article publishing charge ("APC") for Open Access articles. Where the Submitting Author wishes to make the Work available on an Open Access basis (and intends to pay the relevant APC), the terms of reuse of such Open Access shall be governed by a Creative Commons licence – details of these licences and which Creative Commons licence will apply to this Work are set out in our licence referred to above.

Other than as permitted in any relevant BMJ Author's Self Archiving Policies, I confirm this Work has not been accepted for publication elsewhere, is not being considered for publication elsewhere and does not duplicate material already published. I confirm all authors consent to publication of this Work and authorise the granting of this licence.

For peer review only

Does probe-tube verification of real-ear hearing aid amplification characteristics improve outcomes in adult hearing aid users? A protocol for a systematic review

Authors: Ibrahim Almufarrij[1, 2], Kevin J Munro[1, 3] and Harvey Dillon[1,4]

Author affiliations

[1] Manchester Centre for Audiology and Deafness, School of Health Sciences, University of Manchester and the Manchester Academic Health Science Centre, UK

[2] Department of Rehabilitation Sciences, College of Applied Medical Sciences, King Saud University, Riyadh, Kingdom of Saudi Arabia

[3] Manchester University Hospitals NHS Foundation Trust, Manchester Academic Health Science Centre, UK

[4] Macquarie University, Sydney, Australia

Corresponding author: Ibrahim Almufarrij, Manchester Centre for Audiology and Deafness, University of Manchester, Manchester, M13 9PL, United Kingdom. E-mail: Email: ialmufarrij@ksu.edu.sa

For peer review only

ABSTRACT

Introduction: Using a probe-tube microphone to measure and adjust the real-ear performance of the hearing aid to match the prescription target is recommended and widely used in clinical practice. Hearing aid fitting software can approximately match the amplification characteristics of the hearing aid to the prescription without real-ear measurements (REM), but using REM improves the match to the prescribed target. What is unclear is if the improved match results in a better patient-reported outcome. The primary objective of this review is to determine whether the use of REM improves patient-reported outcomes in adult hearing aid users.

Methods and analysis: The review's methods are in accordance with Preferred Reporting Items for Systematic Reviews and Meta-Analyses (PRISMA-P) guidelines. MEDLINE, EMBASE, PsycINFO, CINAHL, Web of Science and CENTRAL via Cochrane Library will be searched to identify relevant studies. The review's population of interest will include adults with any degree of sensorineural or mixed hearing loss who have been prescribed with acoustic hearing aids. The included studies should compare REM fitting to the initial fit provided by the manufacturer's fitting software. Hearing-specific health-related quality of life is the primary outcome but secondary outcomes include self-reported listening ability, speech recognition scores, generic health-related quality of life, hours of use, number of required follow-up sessions and adverse events. Randomised and non-randomised controlled trials will be included. The risk of bias in the included studies will be evaluated using Down and Black's checklist. The quality of the overall evidence will be assessed using the Grading of Recommendations, Assessment, Development and Evaluations (GRADE) tool.

Ethics and dissemination: Ethical approval will not be sought because this systematic review will only retrieve and analyse data from published studies. Review results will be published in a peer-reviewed journal and presented at relevant scientific conferences.

PROSPERO registration number: CRD42020166074

For peer review only

Strengths and limitations of the study:

- This review will be first to synthesise data that compare two commonly used approaches to fit hearing aids to prescription targets (i.e. initial-fit and real-ear measurements).
- A variety of outcome measures will be used to compare these two approaches.
- The implications of this review are important for the emerging category of over-the-counter and direct-to-consumer hearing aids that do not involve real-ear measures.
- We will include only studies that compare these fitting approaches among adults with sensorineural hearing loss, because children and those with other types of hearing loss may require different amplification characteristics.
- Grey literature will not be included (i.e. conference abstracts and theses).

INTRODUCTION

Hearing loss is the most prevalent sensory deficit, affecting more than a fifth of the world's population[1 2]. Untreated hearing loss reduces peoples' ability to communicate effectively, which could lead to social isolation, depression and decreased health-related quality of life[3]. Hearing loss is associated with an increased risk of cognitive decline and dementia but causality is unknown[4]. The rate of unemployment among individuals with hearing loss is higher than that of the general population, costing the UK economy about £25 billion annually in terms of lost economic output[5].

The most prevalent type of hearing loss in adults is sensorineural loss, accounting for more than 90% of all adults with hearing loss in the UK[6]. The primary intervention for permanent hearing loss is the use of acoustic hearing aids[7]. These devices are designed to restore audibility of low-level sounds, maximise intelligibility of conversational level sounds, and maintain comfortability for loud sounds[8]. Hearing aids reduce the handicap caused by mild and moderate hearing loss (e.g. decreased health-related quality of life)[9].

In the UK, hearing aids are fitted and prescribed by state-registered hearing health professionals. The selection of the most appropriate hearing aid is based on a combination of audiometric and non-audiometric factors (e.g., hearing threshold levels, type of hearing loss, physical capabilities of the patient and patient preference, respectively). The optimal

For peer review only

amplification characteristics for each hearing aid user are specified according to validated prescription formulae (e.g. National Acoustic Laboratories Non-Linear 2)[10], which takes into account the patient-specific data (e.g. gender, language and type and degree of hearing loss) to personalise hearing aid output. Modern hearing aid software can approximate the amplification characteristics and the use of this approach for hearing aid fitting is known as initial-fit. A real-ear measurement (REM), which is a tool that can be used to accurately measure the output of a hearing aid when it is coupled to the individual's ear via a soft probe-tube microphone[8], can also be used to guide the adjustment of the output of the hearing aid so that it matches the proposed amplification target that was provided by the validated prescription formula.

Numerous studies have shown that the amplification characteristics approximated by the hearing aid software are inaccurate and significantly deviate from prescription targets[11-13]. These studies also suggest that the use of REMs can improve matches with prescription targets. Given the available evidence, using REMs, to verify the output of hearing aids and achieve better matches with prescription targets, has been endorsed by the British Society of Audiology[14] and most international hearing organisations (e.g. American Speech-Language-Hearing Association)[15]. Nevertheless, it remains unclear if improved matching to prescription targets result in better outcomes.

Using REMs to match hearing aid output to prescription targets requires valuable clinical time, which could otherwise be used to deliver alternative health and hearing services (e.g., identifying and addressing other hearing problems).

Rationale

There is no published systematic review examining the evidence on whether the use of REM improves patient-reported outcomes in adult hearing aid users. Determining the effectiveness of hearing aids fitted using REM would help decision-makers and stakeholders either to continue recommending or to abandon routine use of REM during adult hearing aid fittings. This is an urgent knowledge gap for both stakeholders and clinicians, given that the cost of the REM system is relatively high and it consumes a large amount of clinicians' valuable time. The findings have implications for the emerging category of over-the-counter or direct-to-consumer hearing aids. If fitting hearing aids using REM does not benefit patients, then one of the potential obstacles to people fitting their own hearing aids is overcome. However,

For peer review only

if it is found to be beneficial, decision-makers should continue recommending the use of REMs and poorer outcomes will be expected for direct-to-consumer hearing aids.

Objectives

The objective of this review is to systematically evaluate the current evidence on whether the use of REMs to match the hearing aid's output to one of the validated prescription targets improves outcomes in adult hearing aid users.

METHOD AND ANALYSIS

The protocol of this systematic review has been pre-registered with the International Prospective Register of Systematic Reviews (PROSPERO; CRD42020166074). The systematic review's methods are reported in accordance with Preferred Reporting Items for Systematic review and Meta-Analyses (PRISMA-P) guidelines[16].

Eligibility criteria

The inclusion and exclusion criteria for studies are reported in accordance with participants, interventions, comparators, outcomes and study designs (PICOS) elements.

Participants

Participants will include adults (≥ 18 years old) with any specified degree of sensorineural or mixed hearing loss. Studies that report only a qualitative description of age and threshold of hearing will also be included. If both children and adults are included in a clinical trial, the study will be excluded unless the data were interpreted and reported independently. Participants with other types of hearing loss (i.e. conductive or fluctuating hearing loss) will be excluded.

Interventions

The intervention that will be included is conventional acoustic hearing aids that were programmed to a prescription target using a real-ear measurement system. Assistive listening devices, hearables, personal sound amplification products, and direct-to-consumer hearing devices will be excluded. Implantable devices (e.g. cochlear implants), bone conduction hearing aids or contralateral routing of sounds hearing aids will also be excluded.

For peer review only

Comparators

The comparisons of interest are hearing aids that are programmed to the manufacturers' approximation of the wearers hearing loss without REMs (i.e. initial fit approach).

Outcomes

The primary outcome of interest is hearing-specific health-related quality of life (e.g. Hearing Handicap Inventory for the Elderly)[17]. Secondary outcomes of interest are self-reported listening ability (e.g. Abbreviated Profile of Hearing Aid Benefit)[18], general benefit of hearing aids (e.g. International Outcomes Inventory for Hearing Aids)[19], speech recognition in quiet or noisy environments, generic health-related quality of life, hours of use per day, number of required follow-up care sessions (i.e. for further fine-tuning) and adverse events (e.g. noise-induced hearing loss).

Study designs

Randomised controlled trials and non-randomised controlled trials will be included. Case reports, conference abstracts, book chapters, dissertations, theses, reviews and clinical guidelines will be excluded.

Information sources

Studies that meet the aforementioned eligibility criteria will be identified using a systematic search strategy of the following databases: MEDLINE, EMBASE, PsycINFO, CINAHL, Web of Science and CENTRAL via Cochrane Library. No search restrictions will be applied in terms of the publication's language, status and year.

The reference lists of the included publications will be manually scanned to identify further studies. Using Google Scholar "cited by" feature, publications that have cited any of the included studies will be screened to identify additional relevant articles. Prior to analysis, the searches will be repeated to identify any other relevant studies if there is a significant delay between searches and a manuscript's submission for publication (> 6 months).

For peer review only

Search strategy

The search protocol and methods will be developed by a medical information specialist from Systematic Review Solutions Limited. The included search terms will be based on experts' opinion, free text and controlled terms from Medical Subject Headings (MeSH), Excerpta Medica Tree (EMTREE) and CINAHL headings. The search terms will include many free text terms because truncations may not work well with phrases (e.g. Hearing difficult* might not capture Hearing difficulties). The search strategies for all databases are reported in Appendix 1.

Study records

Data management

Search results, including titles, authors' details, publication years, publication journals and abstracts, will be extracted to EndNote X9 Reference Management software (Clarivate Analytics, 2018). The same software will also be used to remove any duplicates prior to the initial screening. Next, IA will export the title and abstracts of all identified articles into an Excel spreadsheet so that they can be easily screened against the eligibility criteria. The reason for any article's exclusion can also be documented and assessed by KJM and HD. Each article will be assigned a unique number that is linked to the full details of the article.

Selection process

The title and abstract of all identified studies will be screened independently by IA and KJM to determine eligibility for inclusion. A further inspection will be used when there is a discrepancy between the two investigators; this will include assessing the full article. Any disagreement will be resolved by discussion and/or by consulting with the third author (HD). The full text will be retained and inspected by IA and KJM for all articles that match the inclusion criteria. Any disagreements will be resolved via discussion or by consulting the third author. Following PRISMA- recommendations [20], a flow diagram will be used to present the study selection process.

For peer review only

Data collection process and data items

IA and KJM will independently extract the data from the included studies. Should any discrepancies arise, these will be resolved through discussion or consultation with the third author (HD). The data will be extracted in a pre-designed data extraction form adapted from the Cochrane Handbook[21]. The extracted data will include but will not be limited to authors (year), methods, participants, intervention and outcomes (see Table 1). Data presented on graphical forms will be extracted using an online extraction tool (e.g., WebPlotDigitizer; https://automeris.io/WebPlotDigitizer) when necessary.

Table 1 Data items

General information	Authors (year)
	Title
Method	Study design
	Total study duration
	Sequence generation
	Sequence concealment
	Blinding
Participants	Total number
	Country / Setting
	Age
	Sex
	Inclusion and exclusion criteria
Intervention	Intervention group
	Comparator group
Outcome	Primary outcome
	Secondary outcome(s)
Funding source	
Declaration of interest	
Notes	

Risk of bias in individual studies

The assessment of the risk of bias will be conducted independently by IA, KJM and HD. Any disagreements will be resolved using a majority decision. Given the limited number of

For peer review only

randomised controlled trials in the field of audiology, it is anticipated that most of the extracted studies will be non-randomised controlled trials; therefore, the Downs and Black[22] checklist will be used because it is easy to administer, has a well-established validity and reliability and can be used to assess the methodological quality of both randomised and non-randomised studies. Because knowledge of the clinically important differences in hearing aid outcomes is lacking, scoring for the final item (number 27) will be modified based on whether or not the power calculation was performed. That is, one point will be awarded if the calculation was conducted and zero points if it was not. Consequently, the maximum score will be 28. Articles scoring 26–28, 20–25, 15–19 and < 14 will be regarded as having excellent, good, fair and poor quality, respectively. The assessment of the risk of bias will be conducted by the primary and secondary authors independently.

Data synthesis

A meta-analysis will be conducted if enough data are available (i.e. more than one study). If the included studies used the same continuous outcome measures, the mean differences will be calculated with their 95% confidence interval (CI). When different outcome measures are used, the standardised mean difference will be measured along with 95% CI. If the included studies used similar dichotomous outcome measures, the risk ratio will be calculated. If the statistical heterogeneity is low, fixed-effect meta-analyses will be computed; otherwise, only the random-effect meta-analyses will be calculated. For each meta-analysis, the effect size estimate will be calculated using the generic inverse of variance. The effect estimate will be reported along with its 95% CI. Forest plots will be used to present these results. Asymmetrical distribution of continuous outcomes (i.e. skewed data) will be assessed by subtracting the lowest possible value from the mean and then dividing it by the SD. A ratio below 2 or 1 either suggests or indicates a skewed distribution, respectively[23]. Skewed data will be transformed where possible to reduce the skew distribution. Randomised and non-randomised controlled trials will be synthesised separately.

If meta-analysis is not appropriate, the risk of bias of each individual study will be assessed and the findings will be systematically reported

For peer review only

Assessment of reporting bias

The existence of publication bias will be visually inspected using a funnel plot of the study size or precision as a function of intervention effect estimates.

Assessment of heterogeneity

The percentage of variability between studies' outcomes that is due to heterogeneity rather than random error will be computed using an I^2 statistic. Given that the absolute threshold of I^2 is meaningless, the results will be interpreted as either low (0–40%), medium (41–60%) or high (61–100%) heterogeneity.

Dealing with missing data

The corresponding authors will be contacted, where necessary, if any of the data are missing. Data that are only presented in graphical forms will be extracted using the detailed method explained in the section titled Data collection process and data items. In case the standard deviations (SDs) are missing and cannot be obtained from the corresponding authors, they will be estimated from the available data (e.g. 95% CI and standard errors). The reasons for the missing data will be assessed to identify whether they are missing at random or not.

Subgroup analysis

When possible, subgroup analysis will be performed to determine whether age (i.e. ≤ 55 or $>$ 55 years), severity of hearing loss and experience with hearing aids (i.e. first-time vs. experienced users) are possible sources of heterogeneity.

Confidence in cumulative estimate

The quality of each outcome measure will be rated as high, moderate, low or very low using a well-developed assessment tool, the Grading of Recommendations, Assessment, Development and Evaluations tool (GRADE)[24]. The quality rating assigned to each outcome measure reflects our level of confidence as to the veracity of the drawn estimate effect. The GRADE tool takes into account five principal domains: study limitations (e.g. blinding and allocation concealment), inconsistency (e.g., variabilities in effect size), indirectness (e.g. differences in population), imprecision (e.g. broad CI) and publication bias (e.g. selective

For peer review only

reporting of positive outcomes). Randomised controlled trials without serious shortcomings will be rated as high-quality evidence. That is, our confidence level is high enough to conclude that the true effect is close to the drawn estimate effect. Conversely, non-randomised control trials (e.g. observational studies) will be assigned a low rating. In other words, the estimated effect may vary considerably from the true estimate of the effect. However, the assigned rating to randomised and non-randomised controlled trials could be subject to either upgrading or downgrading by either one or two points on the basis of the seriousness of the above-mentioned assessment domains. A thorough discussion of these factors can be found in Schünemann et al (2013)[25]. IA and KJM will carry out the assessment independently and disagreements will be resolved by discussion and/or by consulting HD.

Sensitivity analysis

Sensitivity analysis will be administered to assess the robustness of the pooled estimates. That is, studies with a very high risk of bias (i.e., those that scored less than 14 points on the Downs and Black checklist) will be removed from quantitative synthesis.

Patient and Public Involvement

Patients and the public were not formally involved in the development of the research question and outcomes. The findings will be displayed on the website of The Manchester Center for Audiology and Deafness (ManCAD). In addition, they will be shared with NHS service user groups and Action on Hearing Loss, the largest UK charity for deafness.

ETHICS AND DISSEMINATION

Ethical approval will not be sought because this systematic review will only retrieve and analyse data from published studies. Review results will be published in a peer-reviewed journal, presented at relevant scientific conferences and disseminated on social media.

Contributor IA is the guarantor of this review. IA developed and prepared the review protocol. The search procedure has been developed by a medical information specialist from Systematic Review Solutions Limited. KJM and HD contributed to the development of inclusion and exclusion criteria, search strategy and assessment of the risk of bias. KJM and HD also critically reviewed and provided feedback on earlier versions of the protocol. This manuscript has been approved by all contributors.

For peer review only

Funding NIHR Manchester Biomedical Research Centre (funder reference: IS-BRC-1215-20007)

Competing interest None declared.

ACKNOWLEDGEMENTS

IA, KJM and HD are supported by the NIHR Manchester Biomedical research Centre. The first author is also supported by the Deanship of Scientific Research at the College of Applied Medical Sciences Research Center at King Saud University.

For peer review only

Reference:

1. Mathers C, Smith A, Concha M. Global burden of hearing loss in the year 2000. *Global Burden of Disease* 2000;18:1-30.
2. World Health Organization. Prevention of blindness and deafness: World Health Organization; 2018 [Available from: https://www.who.int/pbd/deafness/estimates/en/ accessed 06/10/2019 2019.
3. Davis A, Smith P, Ferguson M, et al. Acceptability, benefit and costs of early screening for hearing disability: a study of potential screening tests and models. *Health Technol Assess* 2007;11(42):1-294. [published Online First: 2007/10/12]
4. Loughrey DG, Kelly ME, Kelley GA, et al. Association of Age-Related Hearing Loss With Cognitive Function, Cognitive Impairment, and Dementia: A Systematic Review and Meta-analysis. *JAMA Otolaryngol Head Neck Surg* 2018;144(2):115-26. doi: 10.1001/jamaoto.2017.2513 [published Online First: 2017/12/10]
5. International Longevity Centre UK. Commission on Hearing Loss: Final Report, 2014.
6. Action on Hearing Loss. Hearing Matters. UK, 2015.
7. Kochkin S. MarkeTrak VIII: 25-year trends in the hearing health market. *Hearing Review* 2009;16(11):12-31.
8. Dillon H. Hearing Aids: Thieme 2012.
9. Ferguson MA, Kitterick PT, Chong LY, et al. Hearing aids for mild to moderate hearing loss in adults. *Cochrane Db Syst Rev* 2017(9) doi: ARTN CD012023

10.1002/14651858.CD012023.pub2
10. Keidser G, Dillon H, Flax M, et al. The NAL-NL2 Prescription Procedure. *Audiol Res* 2011;1(1):e24. doi: 10.4081/audiores.2011.e24 [published Online First: 2011/05/10]
11. Munro KJ, Puri R, Bird J, et al. Using probe-microphone measurements to improve the match to target gain and frequency response slope, as a function of earmould style, frequency, and input level. *Int J Audiol* 2016;55(4):215-23. doi: 10.3109/14992027.2015.1104736 [published Online First: 2015/11/07]
12. Aazh H, Moore BC. The value of routine real ear measurement of the gain of digital hearing aids. *J Am Acad Audiol* 2007;18(8):653-64. doi: 10.3766/jaaa.18.8.3 [published Online First: 2008/03/11]
13. Aarts NL, Caffee CS. Manufacturer predicted and measured REAR values in adult hearing aid fitting: accuracy and clinical usefulness. *Int J Audiol* 2005;44(5):293-301. doi: 10.1080/14992020500057830 [published Online First: 2005/07/21]
14. British Society of Audiology. Guidance on the verification of hearing devices using probe microphone measurements. Seafield, England: British Society of Audiology, 2018:1 - 32.
15. Valente M, Bentler R, Kaplan HS, et al. Guidelines for hearing aid fitting for adults. . *American Journal of Audiology* 1998;7(1):5 - 13. doi: 10.1044/1059-0889.0701.05.
16. Shamseer L, Moher D, Clarke M, et al. Preferred reporting items for systematic review and meta-analysis protocols (PRISMA-P) 2015: elaboration and explanation. *Bmj* 2015;350:g7647. doi: 10.1136/bmj.g7647 [published Online First: 2015/01/04]
17. Humes LE, Wilson DL, Barlow NN, et al. Changes in hearing-aid benefit following 1 or 2 years of hearing-aid use by older adults. *J Speech Lang Hear Res* 2002;45(4):772-82. doi: 10.1044/1092-4388(2002/062) [published Online First: 2002/08/30]
18. Cox RMA, G. C. The abbreviated profile of hearing aid benefit. *Ear Hear* 1995;16(2):176-86. doi: 10.1097/00003446-199504000-00005 [published Online First: 1995/04/01]
19. Cox RM, Alexander GC. The International Outcome Inventory for Hearing Aids (IOI-HA): psychometric properties of the English version. *Int J Audiol* 2002;41(1):30-5. doi: 10.3109/14992020209101309 [published Online First: 2002/12/07]

For peer review only

20. Moher D, Liberati A, Tetzlaff J, et al. Preferred reporting items for systematic reviews and meta-analyses: the PRISMA statement. *PLoS medicine* 2009;6(7):e1000097. doi: 10.1371/journal.pmed.1000097 [published Online First: 2009/07/22]
21. Higgins JPT, Green S. Cochrane handbook for systematic reviews of interventions. Chichester, England ; Hoboken, NJ: Wiley-Blackwell 2008.
22. Downs SH, Black N. The feasibility of creating a checklist for the assessment of the methodological quality both of randomised and non-randomised studies of health care interventions. *Journal of epidemiology and community health* 1998;52(6):377-84. doi: 10.1136/jech.52.6.377 [published Online First: 1998/10/09]
23. Deeks JJ, Higgins JP, Altman DG. Analysing data and undertaking meta-analyses. In: Higgins JPT, Thomas J, Chandler J, et al., eds. Cochrane Handbook for Systematic Reviews of Interventions. Hoboken: WILEY Blackwell 2019.
24. Atkins D, Best D, Briss PA, et al. Grading quality of evidence and strength of recommendations. *Bmj* 2004;328(7454):1490. doi: 10.1136/bmj.328.7454.1490 [published Online First: 2004/06/19]
25. Schünemann H, Brożek J, Guyatt G, et al. GRADE Handbook2013.

For peer review only

Appendix 1: Search Strategies

A. COCHRANE Library

(("Hearing Loss" OR "hearing loss" OR "hearing losses" OR deaf* OR "Hearing problem" OR "hearing problems" OR hypoacus* OR "hearing impaired" OR "hearing impairment" OR "hearing impairments" OR "hard of hearing" OR "hearing difficulties" OR "hearing difficulty" OR "hearing disabilities" OR "hearing disability" OR "Hearing Disorders" OR "hearing disorder" OR "hearing disorders" OR "hearing loss*" OR "Hearing problem*" OR "hearing impair*" OR "hearing difficult*" OR "hearing disab*" OR "hearing disorder*") AND ("Hearing Aids" OR "hearing aid" OR "hearing aids" OR "hearing device" OR "hearing devices" OR "acoustic aid" OR "acoustic aids" OR "ear molds" OR "ear mold" OR "HA" OR "aid") AND ("real ear measurement" OR "real ear measurements" OR "real ear measure" OR "real ear measures" OR "real-ear measurement" OR "real-ear measurements" OR "real-ear measure" OR "real-ear measures" OR "REM" OR PMM* OR "probe microphone" OR "probe microphones" OR "probe mic" OR "probe mics" OR "probe microphone measurement" OR "probe microphone measurements" OR "probe microphone measure" OR "probe microphone measures" OR "probe-microphone" OR "probe-microphones" OR "probe-microphone measurement" OR "probe-microphone measurements" OR "probe-microphone measure" OR "probe-microphone measures" OR "initial fit" OR "initial fits" OR "initial fitting" OR "initial fittings" OR "real ear" OR "real ears" OR "real-ear" OR "real-ears" OR "hearing aid fit" OR "hearing aid fitter" OR "hearing aid fitting" OR "hearing aid fittings")):ti,ab,kw NOT (("Child" OR "Infant") NOT "Adult*"):ti

B. Embase

(((exp *"Hearing Aid"/ OR "hearing aid".ti OR "hearing aids".ti OR "hearing device".ti OR "hearing devices".ti OR "acoustic aid".ti OR "acoustic aids".ti OR "ear molds".ti OR "ear mold".ti OR "HA".ti OR "aid".ti) AND ("real ear measurement".ti OR "real ear measurements".ti OR "real ear measure".ti OR "real ear measures".ti OR "real-ear measurement".ti OR "real-ear measurements".ti OR "real-ear measure".ti OR "real-ear measures".ti OR "REM".ti OR PMM*.ti OR "probe microphone".ti OR "probe microphones".ti OR "probe mic".ti OR "probe mics".ti OR "probe microphone measurement".ti OR "probe microphone measurements".ti OR "probe microphone measure".ti OR "probe microphone measures".ti OR "probe-microphone".ti OR "probe-microphones".ti OR "probe-microphone measurement".ti OR "probe-microphone measurements".ti OR "probe-microphone measure".ti OR "probe-microphone measures".ti OR "initial fit".ti OR "initial fits".ti OR "initial fitting".ti OR "initial fittings".ti OR "real ear".ti OR "real ears".ti OR "real-ear".ti OR "real-ears".ti OR "hearing aid fit".ti OR "hearing aid fitter".ti OR "hearing aid fitting".ti OR "hearing aid fittings".ti) NOT (exp "juvenile"/ NOT exp "adult"/) NOT (conference review or conference abstract).pt NOT ("case report"/ OR ((exp "review"/ OR exp "Practice Guideline"/ OR "guideline".ti OR "guidelines".ti) NOT exp "Clinical Study"/))) OR ((exp *"Hearing Aid"/ OR "hearing aid".ti,ab OR "hearing

For peer review only

aids".ti,ab OR "hearing device".ti,ab OR "hearing devices".ti,ab OR "acoustic aid".ti,ab OR "acoustic aids".ti,ab OR "ear molds".ti,ab OR "ear mold".ti,ab OR "HA".ti,ab OR "aid".ti,ab) AND ("real ear measurement".mp OR "real ear measurements".mp OR "real ear measure".mp OR "real ear measures".mp OR "real-ear measurement".mp OR "real-ear measurements".mp OR "real-ear measure".mp OR "real-ear measures".mp OR "REM".mp OR PMM*.mp OR "probe microphone".mp OR "probe microphones".mp OR "probe mic".mp OR "probe mics".mp OR "probe microphone measurement".mp OR "probe microphone measurements".mp OR "probe microphone measure".mp OR "probe microphone measures".mp OR "probe-microphone".mp OR "probe-microphones".mp OR "probe-microphone measurement".mp OR "probe-microphone measurements".mp OR "probe-microphone measure".mp OR "probe-microphone measures".mp OR "initial fit".mp OR "initial fits".mp OR "initial fitting".mp OR "initial fittings".mp OR "real ear".mp OR "real ears".mp OR "real-ear".mp OR "real-ears".mp OR "hearing aid fit".mp OR "hearing aid fitter".mp OR "hearing aid fitting".mp OR "hearing aid fittings".mp) AND (exp *"Hearing Impairment"/ OR "hearing loss".ti,ab OR "hearing losses".ti,ab OR deaf*.ti,ab OR "Hearing problem".ti,ab OR "hearing problems".ti,ab OR hypoacus*.ti,ab OR "hearing impaired".ti,ab OR "hearing impairment".ti,ab OR "hearing impairments".ti,ab OR "hard of hearing".ti,ab OR "hearing difficulties".ti,ab OR "hearing difficulty".ti,ab OR "hearing disabilities".ti,ab OR "hearing disability".ti,ab OR "hearing loss*".ti,ab OR "Hearing problem*".ti,ab OR "hearing impair*".ti,ab OR "hearing difficult*".ti,ab OR "hearing disab*".ti,ab OR "hearing disorder*".ti,ab) AND ("randomised".mp OR "randomized".mp OR "nonrandomised".mp OR "nonrandomised".mp OR exp "Randomization"/ OR "Randomized Controlled Trial"/ OR exp "Controlled Clinical Trial"/ OR "Equivalence Trial"/ OR "Pragmatic Trial"/ OR "quasi experimental study"/ OR "Random Allocation".mp OR "Controlled Clinical Trial".mp OR "Controlled Clinical Trials".mp OR "Controlled Trial".mp OR "Controlled Trials".mp OR "Equivalence Trial".mp OR "Pragmatic Clinical Trial".mp OR "Equivalence Trials".mp OR "Pragmatic Clinical Trials".mp OR "RCT".mp OR "RCTs".mp OR "Quasi-Experimental".mp OR "Quasi-Experiment*".mp OR "QuasiExperimental".mp OR QuasiExperiment*.mp OR "Crossover Procedure"/ OR "Cross-Over".mp OR "CrossOver".mp OR "retrospective".mp OR Retrospectiv*.mp OR "Retrospective Study"/ OR "Prospective Study"/ OR "prospective".mp OR prospectiv*.mp OR exp "Evaluation Study"/ OR "evaluate".mp OR evaluat*.mp OR exp "Comparative Study"/ OR "compare".mp OR "comparative".mp OR compar*.mp) NOT (exp "juvenile"/ NOT exp "adult"/) NOT (conference review or conference abstract).pt NOT ("case report"/ OR ((exp "review"/ OR exp "Practice Guideline"/ OR "guideline".ti OR "guidelines".ti) NOT exp "Clinical Study"/))))

C. Emcare

(((exp *"Hearing Aid"/ OR "hearing aid".ti OR "hearing aids".ti OR "hearing device".ti OR "hearing devices".ti OR "acoustic aid".ti OR "acoustic aids".ti OR "ear molds".ti OR "ear mold".ti OR "HA".ti OR "aid".ti) AND ("real ear measurement".ti OR "real ear measurements".ti OR "real ear measure".ti OR "real ear measures".ti OR "real-ear measurement".ti OR "real-ear measurements".ti OR "real-ear measure".ti OR "real-ear

measures".ti OR "REM".ti OR PMM*.ti OR "probe microphone".ti OR "probe microphones".ti OR "probe mic".ti OR "probe mics".ti OR "probe microphone measurement".ti OR "probe microphone measurements".ti OR "probe microphone measure".ti OR "probe microphone measures".ti OR "probe-microphone".ti OR "probe-microphones".ti OR "probe-microphone measurement".ti OR "probe-microphone measurements".ti OR "probe-microphone measure".ti OR "probe-microphone measures".ti OR "initial fit".ti OR "initial fits".ti OR "initial fitting".ti OR "initial fittings".ti OR "real ear".ti OR "real ears".ti OR "real-ear".ti OR "real-ears".ti OR "hearing aid fit".ti OR "hearing aid fitter".ti OR "hearing aid fitting".ti OR "hearing aid fittings".ti) NOT (exp "juvenile"/ NOT exp "adult"/) NOT (conference review or conference abstract).pt NOT ("case report"/ OR ((exp "review"/ OR exp "Practice Guideline"/ OR "guideline".ti OR "guidelines".ti) NOT exp "Clinical Study"/))) OR ((exp *"Hearing Aid"/ OR "hearing aid".ti,ab OR "hearing aids".ti,ab OR "hearing device".ti,ab OR "hearing devices".ti,ab OR "acoustic aid".ti,ab OR "acoustic aids".ti,ab OR "ear molds".ti,ab OR "ear mold".ti,ab OR "HA".ti,ab OR "aid".ti,ab) AND ("real ear measurement".mp OR "real ear measurements".mp OR "real ear measure".mp OR "real ear measures".mp OR "real-ear measurement".mp OR "real-ear measurements".mp OR "real-ear measure".mp OR "real-ear measures".mp OR "REM".mp OR PMM*.mp OR "probe microphone".mp OR "probe microphones".mp OR "probe mic".mp OR "probe mics".mp OR "probe microphone measurement".mp OR "probe microphone measurements".mp OR "probe microphone measure".mp OR "probe microphone measures".mp OR "probe-microphone".mp OR "probe-microphones".mp OR "probe-microphone measurement".mp OR "probe-microphone measurements".mp OR "probe-microphone measure".mp OR "probe-microphone measures".mp OR "initial fit".mp OR "initial fits".mp OR "initial fitting".mp OR "initial fittings".mp OR "real ear".mp OR "real ears".mp OR "real-ear".mp OR "real-ears".mp OR "hearing aid fit".mp OR "hearing aid fitter".mp OR "hearing aid fitting".mp OR "hearing aid fittings".mp) AND (exp *"Hearing Impairment"/ OR "hearing loss".ti,ab OR "hearing losses".ti,ab OR deaf*.ti,ab OR "Hearing problem".ti,ab OR "hearing problems".ti,ab OR hypoacus*.ti,ab OR "hearing impaired".ti,ab OR "hearing impairment".ti,ab OR "hearing impairments".ti,ab OR "hard of hearing".ti,ab OR "hearing difficulties".ti,ab OR "hearing difficulty".ti,ab OR "hearing disabilities".ti,ab OR "hearing disability".ti,ab OR "hearing loss*".ti,ab OR "Hearing problem*".ti,ab OR "hearing impair*".ti,ab OR "hearing difficult*".ti,ab OR "hearing disab*".ti,ab OR "hearing disorder*".ti,ab) AND ("randomised".mp OR "randomized".mp OR "nonrandomised".mp OR "nonrandomised".mp OR exp "Randomization"/ OR "Randomized Controlled Trial"/ OR exp "Controlled Clinical Trial"/ OR "Equivalence Trial"/ OR "Pragmatic Trial"/ OR "quasi experimental study"/ OR "Random Allocation".mp OR "Controlled Clinical Trial".mp OR "Controlled Clinical Trials".mp OR "Controlled Trial".mp OR "Controlled Trials".mp OR "Equivalence Trial".mp OR "Pragmatic Clinical Trial".mp OR "Equivalence Trials".mp OR "Pragmatic Clinical Trials".mp OR "RCT".mp OR "RCTs".mp OR "Quasi-Experimental".mp OR "Quasi-Experiment*".mp OR "QuasiExperimental".mp OR QuasiExperiment*.mp OR "Crossover Procedure"/ OR "Cross-Over".mp OR "CrossOver".mp OR "retrospective".mp OR Retrospectiv*.mp OR "Retrospective Study"/ OR "Prospective Study"/ OR "prospective".mp OR prospectiv*.mp OR exp "Evaluation Study"/ OR "evaluate".mp OR evaluat*.mp OR exp "Comparative Study"/ OR "compare".mp OR "comparative".mp OR

For peer review only

compar*.mp) NOT (exp "juvenile"/ NOT exp "adult"/) NOT (conference review or conference abstract).pt NOT ("case report"/ OR ((exp "review"/ OR exp "Practice Guideline"/ OR "guideline".ti OR "guidelines".ti) NOT exp "Clinical Study"/))))

D. MEDLINE

(("Hearing Loss"/ OR "hearing loss".mp OR "hearing losses".mp OR deaf*.mp OR "Hearing problem".mp OR "hearing problems".mp OR hypoacus*.mp OR "hearing impaired".mp OR "hearing impairment".mp OR "hearing impairments".mp OR "hard of hearing".mp OR "hearing difficulties".mp OR "hearing difficulty".mp OR "hearing disabilities".mp OR "hearing disability".mp OR exp "Hearing Disorders"/ OR "hearing disorder".mp OR "hearing disorders".mp OR hearing loss*.mp OR Hearing problem*.mp OR hearing impair*.mp OR hearing difficult*.mp OR hearing disab*.mp OR hearing disorder*.mp) AND (exp "Hearing Aids"/ OR "hearing aid".mp OR "hearing aids".mp OR "hearing device".mp OR "hearing devices".mp OR "acoustic aid".mp OR "acoustic aids".mp OR "ear molds".mp OR "ear mold".mp OR "HA".ti,ab OR "aid".mp) AND ("real ear measurement".mp OR "real ear measurements".mp OR "real ear measure".mp OR "real ear measures".mp OR "real-ear measurement".mp OR "real-ear measurements".mp OR "real-ear measure".mp OR "real-ear measures".mp OR "REM".mp OR PMM*.mp OR "probe microphone".mp OR "probe microphones".mp OR "probe mic".mp OR "probe mics".mp OR "probe microphone measurement".mp OR "probe microphone measurements".mp OR "probe microphone measure".mp OR "probe microphone measures".mp OR "probe-microphone".mp OR "probe-microphones".mp OR "probe-microphone measurement".mp OR "probe-microphone measurements".mp OR "probe-microphone measure".mp OR "probe-microphone measures".mp OR "initial fit".mp OR "initial fits".mp OR "initial fitting".mp OR "initial fittings".mp OR "real ear".mp OR "real ears".mp OR "real-ear".mp OR "real-ears".mp OR "hearing aid fit".mp OR "hearing aid fitter".mp OR "hearing aid fitting".mp OR "hearing aid fittings".mp) AND ("randomised".mp OR "randomized".mp OR "nonrandomised".mp OR "nonrandomised".mp OR "Random Allocation"/ OR exp "Randomized Controlled Trial"/ OR exp "Randomized Controlled Trials as Topic"/ OR "Non-Randomized Controlled Trials as Topic"/ OR exp "Controlled Clinical Trial"/ OR exp "Equivalence Trial"/ OR exp "Pragmatic Clinical Trial"/ OR exp "Equivalence Trials as Topic"/ OR exp "Pragmatic Clinical Trials as Topic"/ OR "Random Allocation".mp OR "Controlled Clinical Trial".mp OR "Controlled Clinical Trials".mp OR "Controlled Trial".mp OR "Controlled Trials".mp OR "Equivalence Trial".mp OR "Pragmatic Clinical Trial".mp OR "Equivalence Trials".mp OR "Pragmatic Clinical Trials".mp OR "RCT".mp OR "RCTs".mp OR "Quasi-Experimental".mp OR Quasi-Experiment*.mp OR "QuasiExperimental".mp OR QuasiExperiment*.mp OR "Cross-Over Studies"/ OR "Cross-Over".mp OR "CrossOver".mp OR "retrospective".mp OR Retrospectiv*.mp OR exp "Retrospective Studies"/ OR "Prospective Studies"/ OR "prospective".mp OR prospectiv*.mp OR exp "Evaluation Study"/ OR exp "Evaluation Studies as Topic"/ OR "evaluate".mp OR evaluat*.mp OR exp "Comparative Study"/ OR "compare".mp OR "comparative".mp OR compar*.mp) NOT ((exp "Child"/ OR exp "Infant"/) NOT exp "Adult"/) NOT (("Case Reports"/ OR "case report".ti OR "Review"/ OR

"review".ti OR exp "Guideline"/ OR "guideline".ti OR "guidelines".ti) NOT (exp "Clinical Study"/ OR "trial".ti OR "RCT".ti)))

E. PsycINFO

(TX("Hearing Loss" OR "hearing loss" OR "hearing losses" OR deaf* OR "Hearing problem" OR "hearing problems" OR hypoacus* OR "hearing impaired" OR "hearing impairment" OR "hearing impairments" OR "hard of hearing" OR "hearing difficulties" OR "hearing difficulty" OR "hearing disabilities" OR "hearing disability" OR "Hearing Disorders" OR "hearing disorder" OR "hearing disorders" OR "hearing loss*" OR "Hearing problem*" OR "hearing impair*" OR "hearing difficult*" OR "hearing disab*" OR "hearing disorder*") AND TX("Hearing Aids" OR "hearing aid" OR "hearing aids" OR "hearing device" OR "hearing devices" OR "acoustic aid" OR "acoustic aids" OR "ear molds" OR "ear mold" OR "HA" OR "aid") AND TX("real ear measurement" OR "real ear measurements" OR "real ear measure" OR "real ear measures" OR "real-ear measurement" OR "real-ear measurements" OR "real-ear measure" OR "real-ear measures" OR "REM" OR PMM* OR "probe microphone" OR "probe microphones" OR "probe mic" OR "probe mics" OR "probe microphone measurement" OR "probe microphone measurements" OR "probe microphone measure" OR "probe microphone measures" OR "probe-microphone" OR "probe-microphones" OR "probe-microphone measurement" OR "probe-microphone measurements" OR "probe-microphone measure" OR "probe-microphone measures" OR "initial fit" OR "initial fits" OR "initial fitting" OR "initial fittings" OR "real ear" OR "real ears" OR "real-ear" OR "real-ears" OR "hearing aid fit" OR "hearing aid fitter" OR "hearing aid fitting" OR "hearing aid fittings") AND TX("randomised" OR "randomized" OR "nonrandomised" OR "nonrandomised" OR "Random Allocation" OR "Randomized Controlled Trial" OR "Randomized Controlled Trials as Topic" OR "Non-Randomized Controlled Trials as Topic" OR "Controlled Clinical Trial" OR "Equivalence Trial" OR "Pragmatic Clinical Trial" OR "Equivalence Trials as Topic" OR "Pragmatic Clinical Trials as Topic" OR "Random Allocation" OR "Controlled Clinical Trial" OR "Controlled Clinical Trials" OR "Controlled Trial" OR "Controlled Trials" OR "Equivalence Trial" OR "Pragmatic Clinical Trial" OR "Equivalence Trials" OR "Pragmatic Clinical Trials" OR "RCT" OR "RCTs" OR "Quasi-Experimental" OR Quasi-Experiment* OR "QuasiExperimental" OR QuasiExperiment* OR "Cross-Over Studies" OR "Cross-Over" OR "CrossOver" OR "retrospective" OR Retrospectiv* OR "Retrospective Studies" OR "Prospective Studies" OR "prospective" OR prospectiv* OR "Evaluation Study" OR "Evaluation Studies as Topic" OR "evaluate" OR evaluat* OR "Comparative Study" OR "compare" OR "comparative" OR compar*) NOT TI(("Child" OR "Infant") NOT "Adult") NOT TI(("Case Reports" OR "case report" OR "Review" OR "review" OR "Guideline" OR "guideline" OR "guidelines") NOT ("Clinical Study" OR "trial" OR "RCT")))

F. PubMed

(("Hearing Loss"[Mesh] OR "hearing loss"[tw] OR "hearing losses"[tw] OR deaf*[tw] OR "Hearing problem"[tw] OR "hearing problems"[tw] OR hypoacus*[tw] OR "hearing impaired"[tw] OR "hearing impairment"[tw] OR "hearing impairments"[tw] OR "hard of hearing"[tw] OR "hearing difficulties"[tw] OR "hearing difficulty"[tw] OR "hearing disabilities"[tw] OR "hearing disability"[tw] OR "Hearing Disorders"[mesh] OR "hearing disorder"[tw] OR "hearing disorders"[tw] OR hearing loss*[tw] OR Hearing problem*[tw] OR hearing impair*[tw] OR hearing difficult*[tw] OR hearing disab*[tw] OR hearing disorder*[tw]) AND ("Hearing Aids"[Mesh] OR "hearing aid"[tw] OR "hearing aids"[tw] OR "hearing device"[tw] OR "hearing devices"[tw] OR "acoustic aid"[tw] OR "acoustic aids"[tw] OR "ear molds"[tw] OR "ear mold"[tw] OR "HA"[tiab] OR "aid"[tw]) AND ("real ear measurement"[tw] OR "real ear measurements"[tw] OR "real ear measure"[tw] OR "real ear measures"[tw] OR "real-ear measurement"[tw] OR "real-ear measurements"[tw] OR "real-ear measure"[tw] OR "real-ear measures"[tw] OR "REM"[tw] OR PMM*[tw] OR "probe microphone"[tw] OR "probe microphones"[tw] OR "probe mic"[tw] OR "probe mics"[tw] OR "probe microphone measurement"[tw] OR "probe microphone measurements"[tw] OR "probe microphone measure"[tw] OR "probe microphone measures"[tw] OR "probe-microphone"[tw] OR "probe-microphones"[tw] OR "probe-microphone measurement"[tw] OR "probe-microphone measurements"[tw] OR "probe-microphone measure"[tw] OR "probe-microphone measures"[tw] OR "initial fit"[tw] OR "initial fits"[tw] OR "initial fitting"[tw] OR "initial fittings"[tw] OR "real ear"[tw] OR "real ears"[tw] OR "real-ear"[tw] OR "real-ears"[tw] OR "hearing aid fit"[tw] OR "hearing aid fitter"[tw] OR "hearing aid fitting"[tw] OR "hearing aid fittings"[tw]) AND ("randomised"[tw] OR "randomized"[tw] OR "nonrandomised"[tw] OR "nonrandomised"[tw] OR "Random Allocation"[Mesh] OR "Randomized Controlled Trial"[Ptyp] OR "Randomized Controlled Trials as Topic"[Mesh] OR "Non-Randomized Controlled Trials as Topic"[Mesh] OR "Controlled Clinical Trial"[ptyp] OR "Equivalence Trial"[ptyp] OR "Pragmatic Clinical Trial"[ptyp] OR "Equivalence Trials as Topic"[Mesh] OR "Pragmatic Clinical Trials as Topic"[Mesh] OR "Random Allocation"[tw] OR "Controlled Clinical Trial"[tw] OR "Controlled Clinical Trials"[tw] OR "Controlled Trial"[tw] OR "Controlled Trials"[tw] OR "Equivalence Trial"[tw] OR "Pragmatic Clinical Trial"[tw] OR "Equivalence Trials"[tw] OR "Pragmatic Clinical Trials"[tw] OR "RCT"[tw] OR "RCTs"[tw] OR "Quasi-Experimental"[tw] OR Quasi-Experiment*[tw] OR "QuasiExperimental"[tw] OR QuasiExperiment*[tw] OR "Cross-Over Studies"[Mesh] OR "Cross-Over"[tw] OR "CrossOver"[tw] OR "retrospective"[tw] OR Retrospectiv*[tw] OR "Retrospective Studies"[Mesh] OR "Prospective Studies"[Mesh] OR "prospective"[tw] OR prospectiv*[tw] OR "Evaluation Study"[ptyp] OR "Evaluation Studies as Topic"[Mesh] OR "evaluate"[tw] OR evaluat*[tw] OR "Comparative Study"[ptyp] OR "compare"[tw] OR "comparative"[tw] OR compar*[tw]) NOT (("Child"[mesh] OR "Infant"[mesh]) NOT "Adult"[mesh]) NOT (("Case Reports"[ptyp] OR "case report"[ti] OR "Review"[ptyp] OR "review"[ti] OR "Guideline"[Publication Type] OR "guideline"[ti] OR "guidelines"[ti]) NOT ("Clinical Study"[ptyp] OR "trial"[ti] OR "RCT"[ti])))

G. Web of Science

For peer review only

(ts=("Hearing Loss" OR "hearing loss" OR "hearing losses" OR deaf* OR "Hearing problem" OR "hearing problems" OR hypoacus* OR "hearing impaired" OR "hearing impairment" OR "hearing impairments" OR "hard of hearing" OR "hearing difficulties" OR "hearing difficulty" OR "hearing disabilities" OR "hearing disability" OR "Hearing Disorders" OR "hearing disorder" OR "hearing disorders" OR "hearing loss*" OR "Hearing problem*" OR "hearing impair*" OR "hearing difficult*" OR "hearing disab*" OR "hearing disorder*") AND ts=("Hearing Aids" OR "hearing aid" OR "hearing aids" OR "hearing device" OR "hearing devices" OR "acoustic aid" OR "acoustic aids" OR "ear molds" OR "ear mold" OR "HA" OR "aid") AND ts=("real ear measurement" OR "real ear measurements" OR "real ear measure" OR "real ear measures" OR "real-ear measurement" OR "real-ear measurements" OR "real-ear measure" OR "real-ear measures" OR "REM" OR PMM* OR "probe microphone" OR "probe microphones" OR "probe mic" OR "probe mics" OR "probe microphone measurement" OR "probe microphone measurements" OR "probe microphone measure" OR "probe microphone measures" OR "probe-microphone" OR "probe-microphones" OR "probe-microphone measurement" OR "probe-microphone measurements" OR "probe-microphone measure" OR "probe-microphone measures" OR "initial fit" OR "initial fits" OR "initial fitting" OR "initial fittings" OR "real ear" OR "real ears" OR "real-ear" OR "real-ears" OR "hearing aid fit" OR "hearing aid fitter" OR "hearing aid fitting" OR "hearing aid fittings") AND ts=("randomised" OR "randomized" OR "nonrandomised" OR "nonrandomised" OR "Random Allocation" OR "Randomized Controlled Trial" OR "Randomized Controlled Trials as Topic" OR "Non-Randomized Controlled Trials as Topic" OR "Controlled Clinical Trial" OR "Equivalence Trial" OR "Pragmatic Clinical Trial" OR "Equivalence Trials as Topic" OR "Pragmatic Clinical Trials as Topic" OR "Random Allocation" OR "Controlled Clinical Trial" OR "Controlled Clinical Trials" OR "Controlled Trial" OR "Controlled Trials" OR "Equivalence Trial" OR "Pragmatic Clinical Trial" OR "Equivalence Trials" OR "Pragmatic Clinical Trials" OR "RCT" OR "RCTs" OR "Quasi-Experimental" OR Quasi-Experiment* OR "QuasiExperimental" OR QuasiExperiment* OR "Cross-Over Studies" OR "Cross-Over" OR "CrossOver" OR "retrospective" OR Retrospectiv* OR "Retrospective Studies" OR "Prospective Studies" OR "prospective" OR prospectiv* OR "Evaluation Study" OR "Evaluation Studies as Topic" OR "evaluate" OR evaluat* OR "Comparative Study" OR "compare" OR "comparative" OR compar*) NOT ti=(("Child" OR "Infant") NOT "Adult") NOT ti=(("Case Reports" OR "case report" OR "Review" OR "review" OR "Guideline" OR "guideline" OR "guidelines") NOT ("Clinical Study" OR "trial" OR "RCT")) NOT dt=(meeting abstract))

Preferred reporting items for systematic review and meta-analysis protocols (PRISMA-P) 2015

PRISMA-P 2015 Checklist

For peer review only

Section/topic	Item No	Checklist item	Information reported Yes	Information reported No	Page number
**ADMINISTRATIVE INFORMATION**					
**Title**					
Identification	1a	Identify the report as a protocol of a systematic review	☒	☐	1
Update	1b	If the protocol is for an update of a previous systematic review, identify as such	☐	☒	Not an update
**Registration**	2	If registered, provide the name of the registry (e.g., PROSPERO) and registration number in the Abstract	☒	☐	2
**Authors**					
Contact	3a	Provide name, institutional affiliation, and e-mail address of all protocol authors; provide physical mailing address of corresponding author	☒	☐	1
Contributions	3b	Describe contributions of protocol authors and identify the guarantor of the review	☒	☐	11
**Amendments**	4	If the protocol represents an amendment of a previously completed or published protocol, identify as such and list changes; otherwise, state plan for documenting important protocol amendments	☐	☒	Not an amendment
**Support**					
Sources	5a	Indicate sources of financial or other support for the review	☒	☐	12
Sponsor	5b	Provide name for the review funder and/or sponsor	☒	☐	12
Role of sponsor/funder	5c	Describe roles of funder(s), sponsor(s), and/or institution(s), if any, in developing the protocol	☒	☐	12
**INTRODUCTION**					
**Rationale**	6	Describe the rationale for the review in the context of what is already known	☒	☐	4-5
**Objectives**	7	Provide an explicit statement of the question(s) the review will address with reference to participants, interventions, comparators, and outcomes (PICO)	☒	☐	5
**METHODS**					

**Eligibility criteria**	8	Specify the study characteristics (e.g., PICO, study design, setting, time frame) and report characteristics (e.g., years considered, language, publication status) to be used as criteria for eligibility for the review	☒	☐	5-6
**Information sources**	9	Describe all intended information sources (e.g., electronic databases, contact with study authors, trial registers, or other grey literature sources) with planned dates of coverage	☒	☐	6-7
**Search strategy**	10	Present draft of search strategy to be used for at least one electronic database, including planned limits, such that it could be repeated	☒	☐	Appendix 1
STUDY RECORDS					
Data management	11a	Describe the mechanism(s) that will be used to manage records and data throughout the review	☒	☐	7
Selection process	11b	State the process that will be used for selecting studies (e.g., two independent reviewers) through each phase of the review (i.e., screening, eligibility, and inclusion in meta-analysis)	☒	☐	7
Data collection process	11c	Describe planned method of extracting data from reports (e.g., piloting forms, done independently, in duplicate), any processes for obtaining and confirming data from investigators	☒	☐	8
**Data items**	12	List and define all variables for which data will be sought (e.g., PICO items, funding sources), any pre-planned data assumptions and simplifications	☒	☐	7-8
**Outcomes and prioritization**	13	List and define all outcomes for which data will be sought, including prioritization of main and additional outcomes, with rationale	☒	☐	6
**Risk of bias in individual studies**	14	Describe anticipated methods for assessing risk of bias of individual studies, including whether this will be done at the outcome or study level, or both; state how this information will be used in data synthesis	☒	☐	8
DATA					
**Synthesis**	15a	Describe criteria under which study data will be quantitatively synthesized	☒	☐	9
	15b	If data are appropriate for quantitative synthesis, describe planned summary measures, methods of handling data, and methods of combining data from studies, including any planned exploration of consistency (e.g., I^2, Kendall's tau)	☒	☐	9
	15c	Describe any proposed additional analyses (e.g., sensitivity or subgroup analyses, meta-regression)	☒	☐	10-11
	15d	If quantitative synthesis is not appropriate, describe the type of summary planned	☒	☐	9
**Meta-bias(es)**	16	Specify any planned assessment of meta-bias(es) (e.g., publication bias across studies, selective reporting within studies)	☒	☐	10
**Confidence in cumulative evidence**	17	Describe how the strength of the body of evidence will be assessed (e.g., GRADE)	☒	☐	10-11

For peer review only
